# Changes in gene expression during the development of mammary tumors in MMTV-*Wnt-1 *transgenic mice

**DOI:** 10.1186/gb-2005-6-10-r84

**Published:** 2005-09-30

**Authors:** Shixia Huang, Yi Li, Yidong Chen, Katrina Podsypanina, Mario Chamorro, Adam B Olshen, Kartiki V Desai, Anne Tann, David Petersen, Jeffrey E Green, Harold E Varmus

**Affiliations:** 1 Program in Cancer Biology and Genetics, Sloan-Kettering Institute, New York, NY 10021, USA; 2 Breast Center, Baylor College of Medicine, Houston, TX 77030, USA; 3 Department of Medicine, Baylor College of Medicine, Houston, TX 77030, USA; 4 Department of Cell and Molecular Biology, Baylor College of Medicine, Houston, TX 77030, USA; 5 National Human Genome Research Institute, National Institutes of Health, Bethesda, MD 20892, USA; 6 Department of Epidemiology and Biostatistics, Memorial Sloan-Kettering Cancer Center, New York, NY 10021, USA; 7 National Cancer Institute, National Institutes of Health, Bethesda, MD 20892, USA; 8 Johns Hopkins in Singapore Ltd, The Nanos, Singapore 138669, Republic of Singapore

## Abstract

**Background:**

In human breast cancer normal mammary cells typically develop into hyperplasia, ductal carcinoma *in situ*, invasive cancer, and metastasis. The changes in gene expression associated with this stepwise progression are unclear. Mice transgenic for mouse mammary tumor virus (MMTV)-*Wnt-1 *exhibit discrete steps of mammary tumorigenesis, including hyperplasia, invasive ductal carcinoma, and distant metastasis. These mice might therefore be useful models for discovering changes in gene expression during cancer development.

**Results:**

We used cDNA microarrays to determine the expression profiles of five normal mammary glands, seven hyperplastic mammary glands and 23 mammary tumors from MMTV-*Wnt-1 *transgenic mice, and 12 mammary tumors from MMTV-*Neu *transgenic mice. Adipose tissues were used to control for fat cells in the vicinity of the mammary glands. In these analyses, we found that the progression of normal virgin mammary glands to hyperplastic tissues and to mammary tumors is accompanied by differences in the expression of several hundred genes at each step. Some of these differences appear to be unique to the effects of Wnt signaling; others seem to be common to tumors induced by both *Neu *and *Wnt-1 *oncogenes.

**Conclusion:**

We described gene-expression patterns associated with breast-cancer development in mice, and identified genes that may be significant targets for oncogenic events. The expression data developed provide a resource for illuminating the molecular mechanisms involved in breast cancer development, especially through the identification of genes that are critical in cancer initiation and progression.

## Abstract 2

cDNA microarray-derived expression profiles of MMTV-*Wnt-1* and MMTV-*Neu* transgenic mice reveal several hundred genes to be differentially expressed at each stage of breast tumor development.

## Background

Gene expression arrays are being widely used to improve the classification of human cancers and to improve our understanding of the molecular changes associated with carcinogenesis [1,2]. However, their use in defining expression patterns in tumor evolution and in correlating genotypes with phenotypes has been limited because of the poor availability of tissues at different stages in cancer development and because of the great diversity of genetic backgrounds among individuals [3-5]. Mouse models of cancer have advantages for exploring the use of this method: a partially defined neoplastic genotype, relatively uniform genetic background, and ample sources of tissue samples from different stages in mammary tumor evolution. Some features of expression profiles identified in mouse mammary tumors are shared by patterns seen in RNA from human tumors [6]. By comparing expression patterns of mammary tumors in six different transgenic mouse models, Desai and coworkers [7] have shown that the initiating pathway determines a distinctive expression phenotype in tumors. In addition, using proteins as markers of cell phenotypes, we showed that initiating oncogenes determine the developmental status of mammary tumor cells [8].

Members of the *Wnt *gene family were discovered as proto-oncogenes that are frequently activated in mammary tumors arising in mice infected with mouse mammary tumor virus (MMTV) [9,10]. *Wnt *genes encode extracellular matrix binding proteins that control many developmental processes, including cell fate specification and stem cell renewal; they are also involved in mammary morphogenesis and progenitor cell renewal [11,12]. Made as secreted glycoproteins, Wnt proteins exert their biologic effects by binding to at least two membrane receptors, namely the frizzled and low-density lipoprotein receptor related proteins. As a result of signaling via the 'canonical' pathway, β-catenin is stabilized, translocates to the nucleus, and transactivates different sets of genes depending on the cellular context [13].

Mice expressing *Wnt-1 *under the control of the enhancer elements in the MMTV long terminal repeat develop extensive hyperplasias of the mammary glands at prepubertal ages, mammary tumors at a median age of 6 months, and sometimes pulmonary metastases ([14]; Podsypanina K, unpublished observations). Tumors in these MMTV-*Wnt-1 *transgenic mice appear to arise from progenitor cells in the mammary gland, because many cells in both hyperplastic and neoplastic lesions express putative progenitor cell markers (such as Sca-1 and keratin-6) and efflux fluorescent Hoechst 33342 dye - a property that has been associated with stem cells in the hematopoietic system [8,15]. The resulting tumors also contain tumor cells with myoepithelial as well as epithelial markers, implying that they arise from a progenitor cell that gives rise to both lineages [8,15]. Because at least some human breast cancers are also thought to arise from progenitor cells [16], it is important to define better the molecular events that lead to tumor formation in this line of mice.

Here we report the expression profiles at different steps of tumor evolution in the MMTV-*Wnt-1 *transgenic model, and we compare these profiles with those in the MMTV-*Neu *transgenic model. We addressed the following questions. Can we follow progression in MMTV-*Wnt-1 *transgenic mice from hyperplasia to primary tumor? Are differences apparent between tumors induced by different transgenic oncogenes? Can we distinguish tumors with additional genetic alterations in MMTV-*Wnt-1 *transgenic mice from those without other known genetic alterations?

## Results and discussion

### Mammary tumors in MMTV-*Wnt-1 *transgenic mice have an expression profile distinct from that seen in mammary tumors induced by MMTV-*Neu*

Comparison of expression profiles of tumors from several transgenic models has led to the identification of expression signatures for different oncogenic pathways [7]. In order to determine whether tumors from MMTV-*Wnt-1 *transgenic mice also have a distinctive expression profile, we determined the profiles of 23 mammary tumors from MMTV-*Wnt-1 *transgenic mice and, for comparison, 12 mammary tumors from mice carrying the MMTV-*Neu *transgene (Tables 1 and 2 provide sample information and a list of all comparisons). *Neu *(*ErbB2*/*HER2*), a proto-oncogene that is amplified in approximately 25% of human breast cancers [17], encodes a member of the epidermal growth factor receptor family of receptor tyrosine kinases [18]. It activates signaling pathways different from those activated by Wnt-1, and the two oncogenes can collaborate in mammary tumorigenesis [19].

**Figure 1:**
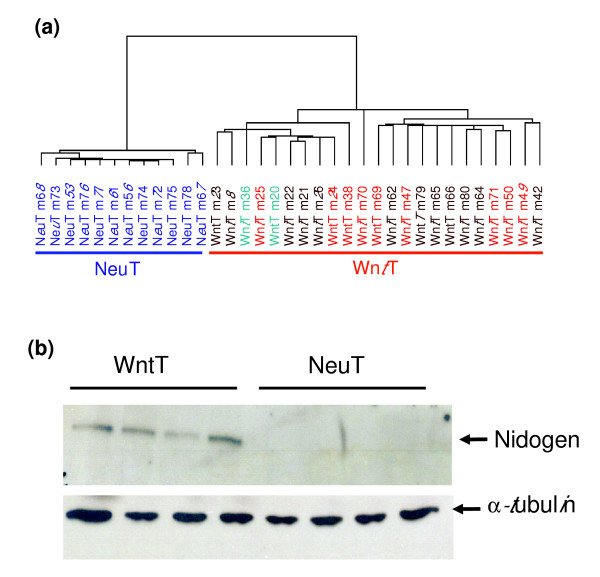
Gene expression in mammary tumors from MMTV-*Wnt-1 *versus MMTV-*Neu *transgenic mice. **(a) **Dendrogram of 35 mammary tumors analyzed by average linkage hierarchical clustering analysis using 1,932 genes selected for high variability across all tumors. 15k arrays were used. The status of *Ha-Ras *on MMTV-*Wnt-1*-induced tumors is color coded: red, wild-type; brown, mutant; green, unknown. **(b) **Western blot analysis for nidogen protein expression on representative mammary tumors from MMTV-*Wnt-1 *and MMTV-*Neu *transgenic mice. MMTV, mouse mammary tumor virus; NeuT, mammary tumors from MMTV-*Neu *transgenic mice; WntT, mammary tumors from MMTV-*Wnt-1 *transgenic mice.

The expression profiles of these two sets of tumors were clearly separated into two groups by unsupervised average linkage hierarchical clustering analysis (Figure 1), suggesting that the global expression patterns of these two sets of tumors differ significantly. This finding extends previous reports of significant divergence in histopathobiology, cellular composition, and possibly the cell types of origin between these two groups of tumors [8,20,21].

In an effort to identify genes that are specifically dysregulated in tumors induced by MMTV-*Wnt-1*, we performed a permutation t-test (see Materials and methods, below, for details) on these two groups of array data. In total, 1,296 genes were differentially expressed between MMTV-*Wnt-1*-induced and MMTV-*Neu*-induced tumors (*P *< 0.001; Table 2 and Additional data file 1). Among the 1,296 genes that we found to be differentially expressed between *Wnt-1*-induced and *Neu*-induced tumors, 842 genes are represented in the 8.7k chips used in the previous report [7]. In that study, 672 genes were found to be differentially expressed among tumors from MMTV-*Neu*, MMTV-*Ha*-*Ras*, MMTV-*c-Myc*, MMTV-*polyoma middle T antigen*, C3(1)/*simian virus 40 T/t antigen*, and Wap-*simian virus 40 T/t antigen *transgenic mice using the 8.7k chips. Comparing the 842 differentially expressed genes in the present study with the 672 genes from the earlier study, we found that 165 genes were present in both lists (Additional data file 1), including 91 of the 178 genes (51%) reported as the Neu-Ras-polyoma middle T antigen cluster [7]. Examples of these 91 genes include *Rap1-GTPase activating protein 1*, *matrix metalloproteinase 15*, and *CD81 *(Additional data file 1).

It should be noted that the MMTV-*Wnt-1 *transgenic mice had a mixed genetic background that was mostly FVB (>75%), whereas MMTV-*Neu *transgenic mice were on a pure FVB background. Although this small variation in genetic background between these two groups of mice is unlikely to account for the differences in expression profiles we detected, we cannot exclude the possibility that some of the genes identified by this analysis might be due to variation in genetic background.

A panel of 652 genes were reported to be differentially expressed between MMTV-*Neu*-induced tumors and normal virgin mammary glands in the study of Desai and coworkers [7] using the 8.7k chips (> two-fold). In the present study comparing 12 tumors from MMTV-*Neu *transgenic mice and five nontransgenic normal virgin mammary glands using the 15k chips, 1,263 genes were differentially expressed (*P *< 0.001, more than three-fold; Table 2). Among these 1,263 genes, 626 genes were represented in the 8.7k arrays used by Desai and coworkers. Of these 626 genes, 225 (35%) overlapped with the 652 genes reported to be differentially expressed between MMTV-*Neu*-induced tumors and normal virgin mammary glands in the study conducted by Desai and colleagues. We consider this to be an acceptable level of reproducibility, considering the multiple differences in the generation of the two data sets (including differences in reference RNAs, array prints, age of the virgin mammary glands, and sample size).

Genes that were more highly expressed (*P *< 0.001) in MMTV-*Wnt-1*-induced tumors than in MMTV-*Neu*-induced tumors include genes reported to be transcriptional targets of Wnt signaling [22-26] such as *cyclin D*_1 _(2.0-fold), *c-Myc *(2.0-fold), *frizzled 7 *(2.1-fold), and *Wnt-5a *(9.2-fold; Additional data file 1). Wnt-5b, another member of the Wnt family, was also more highly expressed (3.7-fold) in tumors from MMTV-*Wnt-1 *transgenic mice than in tumors from MMTV-*Neu *transgenic mice; it remains to be determined whether this Wnt member is also a transcriptional target of Wnt signaling.

Retinoic acid signaling has been reported to synergize with Wnt signaling to induce gene expression [27,28]. *Retinoic acid receptor *and *Stra6*, a gene activated by the addition of retinoids to cultured cells [29], have also been suggested to be targets of Wnt signaling [27]. Consistent with these reports, we found higher level of *Stra6 *(*P *< 0.001, 9.0-fold) in MMTV-*Wnt-1*-induced tumors than in MMTV-*Neu*-induced tumors. In addition, *cellular retinol binding protein *(*RBP*)*1*, a gene related to retinoic acid signaling, was also more highly expressed (*P *< 0.001, two-fold) in MMTV-*Wnt-1*-induced tumors than in MMTV-*Neu*-induced tumors, which is consistent with our recent report that *RBP1 *is induced by β-catenin [30].

MMTV-*Wnt-1*-induced tumors contain both epithelial and myoepithelial cells in approximately equal numbers, unlike tumors induced by the MMTV-*Neu *transgene, which contain only epithelial tumor cells [8,21,31]. Consistent with these reports, we observed higher expression levels (*P *< 0.001) of myoepithelial markers, including *calponin 1 *(12.5-fold) and *calponin 2 *(2.5-fold and 4.0-fold for two separate clones), in tumors from MMTV-*Wnt-1 *transgenic than in tumors from MMTV-*Neu *transgenic mice (Additional data file 1). Consistent with earlier reports that tumors may arise from mammary progenitor cells in MMTV-*Wnt-1 *transgenic mice [8,15], we found that RNA encoding the candidate progenitor cell markers *keratin 6 *(13-fold), *tenascin *(3.1-fold), *osteoblast specific factor 2 *(2.0-fold), *insulin-like growth factor binding protein 7 *(2.0-fold), and *nidogen 1 *(1.8-fold) [8,32] were more abundant (*P *< 0.001) in tumors from MMTV-*Wnt-1 *transgenic mice. Using immunoassays, we demonstrated that keratin 6 and nidogen proteins are expressed at higher level in MMTV-*Wnt-1*-induced tumors than in MMTV-*Neu*-induced tumors (Figure 1b) [8].

### Expression profiles are similar among mammary tumors with additional genetic alterations in MMTV-*Wnt-1 *transgenic mice

The distinct patterns of genes expressed in MMTV-*Wnt-1*-induced and MMTV-*Neu*-induced tumors described in the preceding section suggest that initiating oncogenes strongly influence gene expression in the tumors arising in these two models. We have observed that other genetic events accelerate tumorigenesis in MMTV-*Wnt-1 *transgenic mice [14,33]. We next evaluated whether the events that mediate acceleration are reflected in the gene expression patterns.

We recently reported that approximately 50% of mammary tumors in MMTV-*Wnt-1 *transgenic mice have activating mutations in the *Ha-Ras *locus [19]. Thus, we first considered whether tumors carrying mutant *Ha-Ras *have an expression profile distinct from that observed in tumors that are wild-type at the *Ha-Ras *locus. We sequenced *Ha-Ras *cDNA to seek mutations in 21 out of the 23 MMTV-*Wnt-1*-induced tumors: 12 tumors carry *Ha-Ras *mutations and nine have only *Ha-Ras *wild-type alleles (Figure 1a). Tumors with and without *Ha-Ras *mutations did not have distinct global expression profiles (Figure 1a). Independent multidimensional scaling (MDS) and hierarchical clustering of these 21 tumors based on expression profiles also did not separate them according to *Ha-Ras *status (data not shown). Nevertheless, permutation t test identified 40 genes differentially expressed between tumors bearing wild-type *Ha-Ras *and those carrying a mutant *Ha-Ras *(Table 2 and Additional data file 2). This is more than expected (*P *< 0.001) but many fewer than we saw in our earlier comparison between MMTV-*Wnt-1*-induced and MMTV-*Neu*-induced tumors. In addition, the average fold difference is much smaller (Additional data file 2) than that in the earlier comparison.

**Figure 2:**
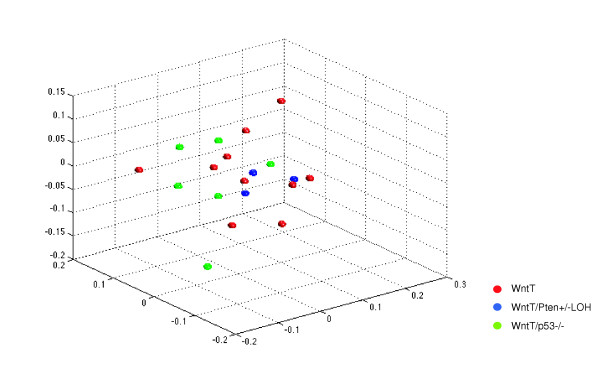
Multidimensional scaling analysis of 18 tumor samples of indicated genotypes. 8.7k arrays were used. MMTV, mouse mammary tumor virus; WntT, mammary tumors from MMTV-*Wnt-1 *transgenic mice; *WntT*/*Pten+/- *loss of heterozygosity (LOH), mammary tumors from MMTV-*Wnt-1 *transgenic/*Pten*^+/- ^mice with *Pten *loss of heterozygosity; WntT/P53-/-, mammary tumors from MMTV-*Wnt-1*/*P53*^-/- ^mice.

We previously determined that loss of either *p53 *or *Pten *accelerates mammary tumorigenesis in MMTV-*Wnt-1 *transgenic mice [34,35]. To further investigate the influence of these genetic alterations on expression patterns in MMTV-*Wnt-1*-induced tumors, we determined the expression profiles of six tumors from MMTV-*Wnt-1 *transgenic mice that were *p53 *null and three tumors from MMTV-*Wnt-1 *transgenic/*Pten*^+/- ^mice that had lost the wild-type allele of *Pten *(i.e. loss of heterozygosity). When genes in the 8.7k array data set from these two groups of tumors and those from 10 tumors from MMTV-*Wnt-1 *transgenic mice that were otherwise wild-type were subjected to analysis by MDS or unsupervised hierarchical clustering, the three groups of samples could not be separated from each other (Figure 2 and data not shown). These findings suggest that the global expression profiles of MMTV-*Wnt-1 *tumors carrying different additional genetic alterations cannot be distinguished.

Permutation t test identified 113 genes that were differentially expressed (p < 0.001) between tumors from MMTV-*Wnt-1 *transgenic mice and those from MMTV-*Wnt-1 *transgenic/*p53*^-/- ^mice (Table 2). Among the 113 genes, 43 were upregulated, and 70 were downregulated in the latter set of tumors (Additional data file 3). Examples of the upregulated genes are *cyclin D*_2 _(3.7-fold), *Myb *(2.9-fold and 2.8-fold for two separate clones), *Bcl11a *(1.5-fold), and *Pbx3 *(1.8-fold average), which promote proliferation or survival. Examples of the downregulated genes are *CD59a antigen *(two-fold), a potential p53 target [36], the *Rb1 *tumor suppressor gene, and the *Met *proto-oncogene. Using similar analyses, we found that 115 genes were differentially expressed (*P *< 0.001) between tumors from MMTV-*Wnt-1 *transgenic mice and those from MMTV-*Wnt-1*/*Pten*^+/- ^mice with *Pten *loss of heterozygosity (Table 2). Forty-five were upregulated, and 70 of them were downregulated in the latter set of tumors (Additional data file 4). Interestingly, among the downregulated genes is *tensin *(two-fold), a cell adhesion molecule that is related to *Pten*. Similar to the comparison between *Ha-Ras *mutant and *Ha-Ras *wild-type tumors in MMTV-*Wnt-1 *transgenic mice, the number of genes differentially expressed and the average fold difference were much smaller in the above two comparisons than in the comparison between MMTV-*Wnt-1*-induced and MMTV-*Neu*-induced tumors (Additional data files 1, 3, and 4).

Collectively, these findings suggest that tumors from MMTV-*Wnt-1 *transgenic mice are similar to each other in their global expression profiles, regardless of whether the tumors have additional genetic alterations. It is not known whether the modest differences in RNA levels we identified among these groups of tumors explain the accelerating effects of these alterations on tumorigenesis in MMTV-*Wnt-1 *transgenic mice. We plan to test some of these changes by expressing cDNAs in mammary glands in *Wnt-1 *transgenic mice using TVA-mediated somatic gene transfer technology [37].

### Distinct changes in gene expression accompany the evolution from normal mammary glands to hyperplasias and to tumors in MMTV-*Wnt-1 *transgenic mice

**Figure 3:**
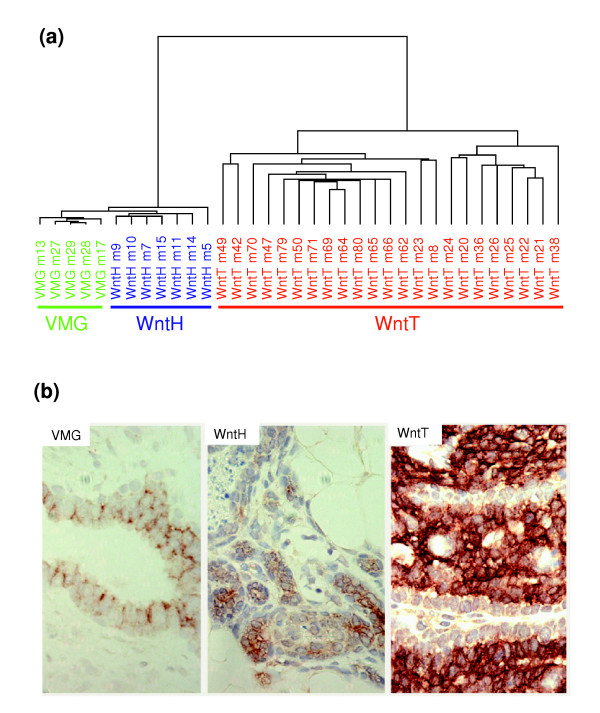
Gene expression in mammary tumor evolution in MMTV-*Wnt-1 *transgenic mice. **(a) **Dendrogram of 35 samples analyzed by average linkage hierarchical clustering analysis, using 3,359 genes selected for high variability across all samples. 15k arrays were used. **(b) **Immunohistochemical staining for c-Kit in the indicated tissue sections. A 20× objective was used. MMTV, mouse mammary tumor virus; VMG, virgin mammary glands from nontransgenic mice; WntT, mammary tumors from MMTV-*Wnt-1 *transgenic mice; WntH, hyperplastic mammary glands from MMTV-*Wnt-1 *transgenic mice.

Hyperplastic lesions are widespread in MMTV-*Wnt-1 *transgenic mice before the development of mammary tumors [14]. To determine whether unique gene expression patterns accompany the evolution from normal mammary cells to hyperplasias and then to tumors, we compared expression profiles among mammary glands of nontransgenic virgin mice, hyperplastic mammary glands, and mammary tumors from MMTV-*Wnt-1 *transgenic mice. Unsupervised hierarchical clustering analysis and MDS showed that expression profiles from these three groups of tissues were separated from each other (Figure 3a and data not shown). The difference between hyperplastic and normal glands is unlikely to be due to decreased contribution of stromal RNA in the preparation of RNAs from the hyperplastic glands from MMTV-*Wnt-1 *transgenic mice, because the expression levels of epithelial and myoepithelial marker genes (*keratin 19*, *calponin 1*, and *calponin 2*) were not significantly statistically different between hyperplastic mammary glands from MMTV-*Wnt-1 *transgenic mice and mammary glands from age-matched nontransgenic virgins.

In total, 584 genes were differentially expressed (*P *< 0.001, Table 1) between hyperplastic mammary glands from MMTV-*Wnt-1 *transgenic mice and normal mammary glands from nontransgenic littermates. Among these 584 genes, 121 were more highly expressed in the hyperplastic glands (Additional data file 5), which includes some of the known transcriptional targets of Wnt signaling such as *c-Myc *(3.6-fold) and *frizzled 7 *(2.1-fold). This list may therefore provide an important starting point for confirming mammary-specific target genes and for discovering novel *in vivo *targets of Wnt signaling. In fact, two genes in this list, namely *RBP1 *(2.9-fold) and *tumor-associated calcium signal transducer *(3.5-fold), were shown to be upregulated by β-catenin in 293 cells in our recent studies [30].

One of the greatest challenges in identifying specific genes and expression patterns associated with the evolution from hyperplastic glands to tumors is the change in cellular composition. Normal and MMTV-*Wnt-1*-induced hyerplastic ductal trees are embedded in stroma, but tumors often contain much less stroma. Thus, the differential contribution of RNA from the stromal cells may skew array analysis, which is based on total RNA content. However, stromal cells are mostly large adipocytes whose RNA to mass ratio is small; thus, the relative contribution of RNA from these cells is probably much less than it appears to be from histologic assessment. The average expression level of epithelial and myoepithelial markers (*keratin 19*, *calponin 1*, and *calponin 2*) was 2.6-fold higher in the tumors (which contain very few stromal cells) than in the hyperplastic tissues, suggesting that 38% (1/2.6 = 38%) of the RNA in the hyperplastic tissues might come from the ducts and alveoli. Thus, to eliminate genes that were not truly differentially expressed, we filtered out any genes that were less than three-fold different in our comparison between tumors and hyperplastic glands in Table 2.

Based on the above calculation, approximately 62% of the RNAs from hyerplastic mammary glands might come from adipocyte-rich stroma. Thus, tumor samples, which have very little contribution from adipocytes, may appear to have downregulated the genes that are associated with adipocytes. In order to identify these genes, we compared the expression profiles of a set of three fat samples with those of the 35 mammary tumors from MMTV-*Wnt-1 *and MMTV-*Neu *transgenic mice. Expression of 741 genes was at least three-fold or higher (*P *< 0.001) in fat than in the mammary tumors (Table 2 and Additional data file 6). These include published fat-specific genes (Additional data file 6), such as *fat-specific gene 27*, *lipoprotein lipase*, *CD36*, *carbonic anhydrases*, and *solute carrier family members *[7]. We note these genes in our table comparing hyperplastic mammary glands with tumors (Additional data file 7).

In total, 1,372 genes were differentially expressed (*P *< 0.001) between tumors and hyperplastic glands from MMTV-*Wnt-1 *transgenic mice. Among them, expression levels for 388 differed by at least three-fold (Additional data file 7). This subgroup is likely to contain genes that are important for the evolution of hyperplastic lesions into tumors, including genes that are required for tumor cell proliferation and survival. One such candidate is *c-Kit*, a proto-oncogene that is frequently overexpressed in cancers and that encodes a receptor that activates both Ras and Akt pathways. The expression of *c-Kit *was 3.6-fold higher in tumors than in hyperplastic lesions (Additional data file 7), although it was similarly expressed in normal virgin glands and hyperplastic mammary glands from MMTV-*Wnt-1 *transgenic mice. This is consistent with a recent report that c-Kit is highly expressed in the basal group of human breast tumors compared to other groups [38]. Using immunohistochemical staining, c-Kit protein was barely detectable in normal mammary glands from nontransgenic mice and in hyperplastic mammary glands from MMTV-*Wnt-1 *transgenic mice, but was readily and widely detected in the tumor samples from MMTV-*Wnt-1 *transgenic mice (Figure 3b).

Some of the genes that were differentially expressed between mammary tumors and hyperplastic glands in MMTV-*Wnt-1 *transgenic mice may be needed for evolution of tumors induced by both Wnt-1 and other oncogenes. Other genes may be uniquely important for induction of tumors from hyperplastic glands in MMTV-*Wnt-1 *transgenic mice. For example, certain signaling pathways may need to be activated in hyperplastic cells in MMTV-*Wnt-1 *transgenic mice before a tumor will form, but they may be optional for tumorigenesis initiated by other oncogenes. To discover genes that might be uniquely important for tumors to develop in hyperplastic glands in MMTV-*Wnt-1 *transgenic mice, we compared the 388 genes that we found to be differentially expressed between tumors and hyperplastic glands from MMTV-*Wnt-1 *transgenic mice with the 1,296 genes that we found to be differentially expressed between tumors from MMTV-*Wnt-1 *transgenic and MMTV-*Neu *transgenic mice. Fifty-six genes corresponding to 59 cDNA clones in the former group were shared in the latter group (Table 3), suggesting they might be specifically involved in neoplastic progression in MMTV-*Wnt-1 *transgenic mice. Among these 56 genes, 23 were more highly expressed and 33 were expressed at lower level in MMTV-*Wnt-1*-induced tumors than in either MMTV-*Wnt-1*-induced hyperplasia or MMTV-*Neu*-induced tumors (Table 3). The upregulated genes (*P *< 0.001) include *TNFRSF19 *(3.5-fold), *NGFR *(3.6-fold), *apolipoprotein D *(4.7-fold), and *Wnt5a *(4.4-fold), and the downregulated genes (*P *< 0.001) include *BNIP3 *(2.5-fold) and *caveolin *(2-, 3.3-, and 3.3-fold for three separate clones). Of note, *apolipoprotein D *has been reported to be upregulated in a subset of human breast cancers [39], and caveolin 1, a negative regulator of the Ras-p42/p44 mitogen-activated protein kinase cascade, has been reported to inhibit growth in human breast cancer cells [40].

## Conclusion

Our analysis of different stages of tumorigenesis in mouse models identified changes in gene expression accompanying tumor initiation and evolution. We also extended the report by Desai and coworkers [7] that the initiating oncogene determines the expression profiles of primary mammary tumors. In addition, we observed that the tumors from MMTV-*Wnt-1 *transgenic mice are similar to each other in their global expression profiles, regardless of whether the tumors have additional genetic alterations. These data may be useful for elucidation of oncogenic signaling pathways in breast cancer initiation and evolution.

## Materials and methods

### cDNA microarray slides

The mouse 15k slides and 8.7k slides used in this study were arrayed at the National Cancer Institute microarray facility. All slides of each array type were printed in a single batch. The 8.7k slides contain the 8700 Incyte GEM1 clone set, which are mapped to 6,877 Unigene cluster IDs, among which 2,953 are named genes, 2,206 are expressed sequence tags, and 1,628 are Riken cDNAs. The 15k slides contain the 8700 Incyte GEM1 clone set and the mammary 6000 clone set; a total of 1,444 clones do not map to a Unigene cluster ID, whereas the rest of the clones map to 10,062 unique genes as defined by Unigene cluster ID. Among the 10,062 Unigene clusters, 3,750 are named genes, 3,922 are expressed sequence taqs, and 2,390 are Riken cDNAs.

### Sample information

All nontransgenic and MMTV-*Neu *transgenic animals used in this study were on the FVB background. All MMTV-*Wnt-1 *transgenic mice [14] were a mixture of FVB (>75%), SJL, and C57BL/6 strains. MMTV-*Neu *transgenic mice [41] were purchased from Jackson Laboratories (Bar Harbor, ME, USA). This transgenic line carries a rat cDNA encoding the wild-type Neu protein. Fat tissues were collected from intestinal fat in virgin FVB mice. All samples were collected fresh and snap-frozen in liquid nitrogen. RNA was extracted by Trizol (Invitorgen, Carlsbad, CA, USA). Reference RNA was a mixture of ovarian RNA (Ambion, Austin, TX, USA; Cat number 7824) and RNA extracted from tissues of liver, spleen, kidney, thymus, pancreas, lung, and normal lactating mammary gland of FVB mice of 6 months of age. All reference RNA used in this study is from a single preparation.

### cDNA microarray hybridization and data extraction

The cDNA probes were prepared from a total of 35-50 μg reference RNA and 50-75 μg sample RNA from normal, hyperplastic, or tumor tissues, as described [42,43]. The cDNA from reference RNA was labeled with cyanine 3-dUTP, and that from sample RNA was labeled with cyanine 5-dUTP. Fluorescent images of hybridized microarrays were obtained by using a GenePix 4000 scanner (Axon Instruments, Foster City, CA, USA). Microarray images were analyzed using the ArraySuite software [44,45] based on the Scanalytics IPlab platform (Scanalytics, Fairfax, VA, USA). For each cDNA probe location, fluorescence intensity ratio and its associated measurement quality (q) were calculated. The evaluation of measurement quality is based on spot size, signal to noise ratio, background uniformity, and saturation pixel percentage [45]. The range of measurement quality is from 1.0 to 0, with higher measurements reflecting better quality. Areas of the array with obvious blemishes were automatically given a low quality value.

### Statistical analysis of cDNA microarray data

Normalized log test to reference ratios and their corresponding quality measurements in each experiment were calculated as described previously [45]. A gene was excluded from further analyses (see below for description) if the average quality measurement was under 0.5 across samples in that specific comparison. Approximately 14,000 genes are suitable for analyzing on the 15k chips and 8,000 genes on the 8.7k chips.

Two methods were used to visualize the expression patterns among samples. Both used the Pearson correlation as a similarity measure. In average linkage hierarchical clustering, the distances between samples are represented on a tree called a dendrogram. In MDS, samples with similar expression ratios were placed closer to each other in three dimensional space. Average linkage hierarchical clustering analysis was implemented using the CLUSTER program, and the results were displayed using TREEVIEW [46]. MDS was developed in the MATLAB (Natick, MA, USA) environment.

A permutation t-test was used to select genes significantly differentially expressed between any two groups [47]. Here, a standard t-statistic was computed between two groups on the log-transformed ratios of each gene. The group labels were randomly permuted and the t-statistic for each gene in the permuted data set was calculated. The process was repeated 10,000 times. A *P *value was reported for each gene by comparing the observed statistic with the permutation statistics. To control for multiple comparisons, only genes with *P *values less than 0.001 were considered differentially expressed. The distribution of the significant differences expected by chance and the probability of observing as many or more differentially expressed genes were calculated from the permuted data. This latter probability is the *P *value reported in the Results and discussion section.

### Western blot analysis and immunohistochemical staining

Antibodies used include rabbit IgG directed against nidogen and c-Kit (Santa Cruz, CA, USA). Tissues were fixed in 10% neutral formalin and processed as previously described [34] to obtain paraffin sections of 4 μm in thickness. For immunohistochemistry, the sections were boiled for 15 minutes in 10 mmol/l citrate buffer of pH 6.0 (to unmask antigen epitopes). Endogenous peroxidase activity was inactivated by 10 minute incubation in 3% hydrogen peroxide, and subsequent steps were performed using Vector ABC kits and the Nova-Red substrate (Vector Laboratories, Burlingame, CA, USA) following the manufacturer's recommendations.

For Western blotting, tumors were ground to powder in liquid nitrogen and lysed in the M-PER tissue lysis solution (Pierce, Rockford, IL, USA) with gentle shaking overnight at 4°C. Proteins in resulting supernatant (25 μg protein) were denatured using 2-mercaptoethanol, resolved on 10% polyacrylamide mini-gels containing 10% sodium dodecyl sulfate, and transferred to nitrocellulose membranes. The membranes were then incubated with primary antibodies and peroxidase-conjugated secondary antibodies (Jackson Laboratories) in tris-borate buffer (1 mmol/l Tris and 13.7 mmol/l NaCl, pH 7.6)/0.05% Tween 20/5% nonfat dried milk. Proteins recognized by specific antibodies were visualized using a chemiluminescent substrate (Supersignal; Pierce).

### Accession number

The GEO accession number for the series of array data-sets is GSE2860.

## Additional data files

The following additional data are available with the online version of this article: a table listing genes differentially expressed between mammary tumors from MMTV-*Wnt-1 *and MMTV-*Neu *transgenic mice (Additional data file 1); a table listing genes differentially expressed between *Ha-Ras *mutant and *Ha-Ras *wild-type tumors in MMTV-*Wnt-1 *mice (Additional data file 2); a table listing genes differentially expressed between MMTV-*Wnt-1*-induced tumors in *p53*-null and in *p53*-wild-type background (Additional data file 3); a table listing genes differentially expressed between tumors from MMTV-*Wnt-1 *transgenic/*Pten*^+/- ^mice with loss of heterozygosity at the *Pten *locus and tumors from *Wnt-1 *transgenic/*Pten*^+/+ ^mice (Additional data file 4); a table listing genes differentially expressed between virgin mammary glands from nontransgenic mice and hyperplastic mammary glands from MMTV-*Wnt-1 *transgenic mice (Additional data file 5); a table listing genes expressed three-fold or higher in fat tissues than in mammary tumors from MMMTV-*Wnt-1 *and MMTV-*Neu *transgenic mice (Additional data file 6); and a table listing genes differentially expressed between mammary tumors and hyperplastic mammary glands from MMTV-*Wnt-1 *transgenic mice (Additional data file 7).

## Supplementary Material

Additional File 1 A table listing genes differentially expressed between mammary tumors from MMTV-*Wnt-1 *and MMTV-*Neu *transgenic mice Click here for file

Additional File 2 A table listing genes differentially expressed between *Ha-Ras *mutant and *Ha-Ras *wild-type tumors in MMTV-*Wnt-1 *mice Click here for file

Additional File 3 A table listing genes differentially expressed between MMTV-*Wnt-1*-induced tumors in *p53*-null and in *p53*-wild-type background Click here for file

Additional File 4 A table listing genes differentially expressed between tumors from MMTV-*Wnt-1 *transgenic/*Pten*^+/- ^mice with loss of heterozygosity at the *Pten *locus and tumors from *Wnt-1 *transgenic/*Pten*^+/+ ^mice Click here for file

Additional File 5 A table listing genes differentially expressed between virgin mammary glands from nontransgenic mice and hyperplastic mammary glands from MMTV-*Wnt-1 *transgenic mice Click here for file

Additional File 6 A table listing genes expressed three-fold or higher in fat tissues than in mammary tumors from MMMTV-*Wnt-1 *and MMTV-*Neu *transgenic mice Click here for file

Additional File 7 A table listing genes differentially expressed between mammary tumors and hyperplastic mammary glands from MMTV-*Wnt-1 *transgenic mice Click here for file

## Figures and Tables

**Table 1:** Tissue samples

Tissue type	Abbreviation	Number of samples	Age (weeks)	Array size
Normal virgin mammary gland	VMG or V	5	9	15k
Hyperplastic mammary glands from MMTV-*Wnt-1 *transgenic mice	WntH	7	9	15k
Mammary tumors from MMTV-*Wnt-1 *transgenic mice	WntT	33	9-56	15k (23 arrays), 8.7k (10 arrays)
Mammary tumors from MMTV-*Neu *transgenic mice	NeuT	12	32-60	15k
Normal fat tissue	Fat	3	12	15k
Mammary tumors from MMTV-*Wnt-1 *transgenic/*P53*^-/- ^mice	WntT/p53^-/-^	6	9-14	8.7k
Mammary tumors from MMTV-*Wnt-1 *transgenic/*Pten*^+/- ^mice with LOH at the *Pten *locus	WntT/Pten^+/-^LOH	3	12-21	8.7k

LOH, loss of heterozygosity; MMTV, mouse mammary tumor virus.

**Table 2:** Numbers of genes that are differentially expressed

Comparisons		Number of genes differentially expressed		
Tissue A (number of samples)	Tissue B (number of samples)	Total	Up	Down

WntH (7)	VMG (5)	584	121	463
WntT (23)	WntH (7)	388*	112	276
WntT (23)	NeuT (12)	1,296	624	672
NeuT (12)	VMG (5)	1,263*	419	844
WntT, H-ras mutant (12)	WntT, H-ras wild-type (9)	40	31	9
WntT/p53^-/-^	WntT (10)	113	43	70
WntT/Pten^+/- ^LOH (3)	WntT (10)	115	45	70

Expression ratio is computed by dividing the average expression level of the A group by the average expression level of the B group. The numbers of differentially expressed genes were determined by random permutation (*P *< 0.001), as described in the Materials and method section. *In selected comparisons, to reduce potential false signals due to stromal effects, the genes that were less than three-fold different in expression were filtered out from the listed total number of genes. LOH, loss of heterozygosity.

**Table 3:** List of genes potentially specifically involved in neoplastic progression in MMTV-*Wnt-1 *transgenic mice

			Expression ratio		
			
Image ID	Gene name	Symbol	WntH/V	WntT/WntH	WntT/NeuT
474107	Tumor necrosis factor receptor superfamily, member 19	Tnfrsf19	2.56	3.48	6.23
406897	Retinol binding protein 1, cellular	Rbp1	2.93	3.02	1.96
572428	Cell cycle cyclin D1 Mm.35804 Cyclin D1	Ccnd1	=	5.53	1.97
536306	Hormone/GF growth/diff 1 Mm.22621 Procollagen type I α1	Col1a1	=	3.04	3.27
536526	ESTs		=	8.8	5.95
329780	Cadherin 3	Cdh3	=	3.74	3.96
427360	*Mus musculus*, clone IMAGE:3590270, mRNA, partial cds	LOC192176	=	3.5	2.44
638805	Expressed sequence AI504637		=	3.3	2.27
355990	Chondroitin sulfate proteoglycan 2	Cspg2	=	3.62	5.05
574500	ESTs		=	4.13	1.65
476431	Nerve growth factor receptor	Ngfr	=	3.61	6.34
680894	Glial cell line derived neurotrophic factor family receptor α1	Gfra1	=	4.74	4.05
482943	Odd-skipped related 1 (*Drosophila*)	Osr1	=	4.1	3.65
695687	Calponin 2	Cnn2	=	3.49	2.54
335572	Dihydropyrimidinase-like 3		=	3.27	7.61
621246	Interferon concensus sequence binding protein	Icsbp	=	3.38	4.91
482170	Four jointed box 1 (*Drosophila*)	Fjx1	=	4.13	3.78
468019	IL-17b	Il17b	=	4.27	5.37
373716	Tumor-associated calcium signal transducer 2	Tacstd2	=	4.16	3.69
479405	ESTs		=	3.01	4.95
722262	Wingless-related MMTV integration site 5A	Wnt5a	=	4.44	9.23
719592	GATA binding protein 2	Gata2	=	6.06	10.54
1247541	Apolipoprotein D	Apod	=	4.67	3.86
331186	Caveolin, caveolae protein, 22 kDa	Cav1	0.31	0.29	3.67
596968	Caveolin, caveolae protein, 22 kDa	Cav1	0.3	0.31	3.05
948509	Caveolin, caveolae protein, 22 kDa	Cav1	0.52	0.18	3
386555	CD36 antigen	Cd36	0.21	0.06	0.08
832585	ESTs		0.21	0.06	0.21
620819	transcription elongation factor A (SII) 1	AI158848	0.24	0.07	0.25
831701	ESTs		0.2	0.09	0.25
775253	ESTs		0.16	0.07	0.22
334182	Unknown		0.24	0.06	0.21
493675	Riken cDNA 2700018N07 gene	Scd1	0.18	0.1	0.21
571367	Riken cDNA 2410012F02 gene		0.24	0.11	0.27
463388	Epoxide hydrolase 2, cytoplasmic	Ephx2	0.3	0.11	0.41
374030	EST		0.25	0.11	0.33
1067881	Fc receptor, IgG, low affinity III	Fcgr3	0.36	0.16	0.26
474184	Expressed sequence AW554339		0.3	0.15	0.33
579349	Expressed sequence AI593221	2410127E18Rik	0.29	0.18	0.23
874232	Riken cDNA 1110025G12 gene	1110025G12Rik	0.33	0.19	0.56
891453	Expressed sequence AI315208	AI315208	=	0.2	0.16
1399595	Riken cDNA 2810422B09 gene	1810061M12Rik	0.3	0.18	0.4
737745	ESTs		0.34	0.17	0.37
876063	BCL2/adenovirus E1B 19 kDa-interacting protein 1, NIP3	Bnip3	0.38	0.27	0.62
850642	Epoxide hydrolase 2, cytoplasmic	Ephx2	0.18	0.22	0.44
1248105	Expressed sequence AI595343	AI595343	0.42	0.21	0.37
330661	*Mus musculus *golli-interacting protein mRNA, complete cds	Nif3	0.66	0.21	0.22
1349720	Apoptosis NIP3, Bcl-2-binding protein homolog (Nip3) mRNA	Bnip3	0.37	0.29	0.52
1067414	Expressed sequence AI413399	1110001E17Rik	=	0.24	0.52
832584	ESTs, Weakly similar to STHM MOUSE STATHMIN [*M. musculus*]		0.31	0.21	0.46
876369	Amyotrophic lateral sclerosis 2 (juvenile) homolog (human)	Als2	0.41	0.29	0.39
764542	ESTs, Weakly similar to GPRY_mouse probable G protein-coupled receptor GPR34 [*M. musculus*]	Gpr43	0.49	0.24	0.43
1248075	Transcription factor 1	Tcf1	0.29	0.31	0.36
949592	ESTs, Moderately similar to hypothetical protein [*H. sapiens*]		=	0.32	0.22
1195295	Actinin α3	Actn3	=	0.29	0.43
820307	A kinase (PRKA) anchor protein (gravin) 12	Akap12	=	0.31	0.08
864409	Riken cDNA 1200013I08 gene	1200013I08Rik	0.47	0.33	0.39
947659	Riken cDNA 2310016E22 gene	2310016E22Rik	=	0.33	0.37
1396547	Myosin binding protein H		=	0.25	0.45

This list displays genes that are differentially expressed between mammary tumors (WntT) and hyperplastic mammary gland (WntH) from MMTV-*Wnt-1 *transgenic mice, and that are also differentially expressed between WntT and mammary tumors from MMTV-*Neu *transgenic mice (NeuT). Genes are sorted according to the average ratio of WntT versus WntH. A numeric ratio is displayed if the gene expression meets the criteria (statistical significance and fold cutoff) described in Table 2; otherwise, it is marked as '=', indicating that there is no significant difference between the two sets of samples in comparison. EST, expressed sequence tag; MMTV, mouse mammary tumor virus.
