# Supplementary material for: Changes in gene expression during the development of mammary tumors in MMTV-Wnt-1 transgenic mice
Source: Genome Biol. 2005 Sep 30;6(10):R84. doi: 10.1186/gb-2005-6-10-r84 (PMC1257467; doi:10.1186/gb-2005-6-10-r84)
Supplement: Additional File 1 — A table listing genes differentially expressed between mammary tumors from MMTV-Wnt-1 and MMTV-Neu transgenic mice [file gb-2005-6-10-r84-S1.doc]

| **Additional data file 1. List of genes differentially expressed between mammary tumors from MMTV-Wnt-1 and MMTV-Neu TG mice** | | | |
| --- | --- | --- | --- |
| **Image ID** | **Gene Name** | **Symbol** | **Expression Ratio**** |
| 355929 | inhibitor of DNA binding 4 | Idb4 | 72.45 |
| 464060 | keratin complex 1, acidic, gene 19 | Krt1-19 | 55.88 |
| 478168 | pleiotrophin | Ptn | 40.09 |
| 570673 | crystallin, mu | Crym | 17.65 |
| 481011 | tripartite motif protein 29 | Trim29 | 16.97 |
| 672405 | small proline-rich protein 1A | Sprr1a | 16.45 |
| 441290 | tripartite motif protein 29 | Trim29 | 13.75 |
| 335736 | keratin complex 2, basic, gene 6a | Krt2-6a | 13.04 |
| 373793 | calponin 1 | Cnn1 | 12.47 |
| 719592 | GATA binding protein 2 | Gata2 | 10.54 |
| 426146 | transforming growth factor beta 1 induced transcript 1 | Tgfb1i1 | 9.72 |
| 722262 | wingless-related MMTV integration site 5A | Wnt5a | 9.23 |
| 442048 | stimulated by retinoic acid gene 6 | Stra6 | 8.95 |
| 1364726 | chondroitin sulfate proteoglycan 2 | Cspg2 | 8.18 |
| 1447403 | WASP family 1 | Wasf1 | 7.89 |
| 1178410 | procollagen, type XIV, alpha 1 | Col14a1 | 7.72 |
| 335572 | dihydropyrimidinase-like 3 | Dpysl3 | 7.61 |
| 484261 | keratin complex 1, acidic, gene 13 | Krt1-13 | 7.6 |
| 424433 | cyclin D2 | Ccnd2 | 7.37 |
| 621166 | ectonucleotide pyrophosphatase/phosphodiesterase 2 | Enpp2 | 7.22 |
| 418546 | transforming growth factor beta 1 induced transcript 1 | Tgfb1i1 | 7.15 |
| 493238 | lymphocyte antigen 6 complex, locus A | Ly6 | 6.95 |
| 621040 | prostaglandin E synthase | Ptges | 6.88 |
| 481198* | tumor-associated calcium signal transducer 2 | Tacstd2 | 6.87 |
| 440103 | ectonucleotide pyrophosphatase/phosphodiesterase 2 | Enpp2 | 6.73 |
| 643463 | nucleolar protein 5 | Nol5 | 6.65 |
| 863863 | expressed sequence AI115600 | Rcn2 | 6.65 |
| 385744 | expressed sequence AI158848 | AI158848 | 6.63 |
| 888512 | small proline-rich protein 2A | Sprr2a | 6.63 |
| 904900 | myelin and lymphocyte protein, T-cell differentiation protein | Mal | 6.55 |
| 476431 | nerve growth factor receptor | Ngfr | 6.34 |
| 474107 | tumor necrosis factor receptor superfamily, member 19 | Tnfrsf19 | 6.23 |
| 671218 | midkine | Mdk | 6.15 |
| 351947 | leucine rich repeat protein 1, neuronal | Lrrn1 | 6.14 |
| 403869 | crystallin, beta A4 | Cryba4 | 6.02 |
| 1244047 | activated leukocyte cell adhesion molecule | Alcam | 6 |
| 418564 | axin2 | Axin2 | 5.78 |
| 832251 | procollagen, type IV, alpha 1 | Col4a1 | 5.73 |
| 472722 | procollagen, type XIV, alpha 1 | Col14a1 | 5.67 |
| 748587* | histocompatibility 2, class II antigen E beta | H2-Eb1 | 5.65 |
| 717457 | fibroblast growth factor binding protein 1 | Fgfbp1 | 5.59 |
| 536577 | dickkopf homolog 3 (Xenopus laevis) | Dkk3 | 5.58 |
| 662933 | FXYD domain-containing ion transport regulator 2 | Fxyd2 | 5.55 |
| 459700 | GATA binding protein 2 | Gata2 | 5.53 |
| 721906* | S100 calcium binding protein A6 (calcyclin) | S100a6 | 5.44 |
| 468019* | interleukin 17B | Il17b | 5.37 |
| 747939 | growth arrest and DNA-damage-inducible 45 gamma | Gadd45g | 5.22 |
| 355990 | chondroitin sulfate proteoglycan 2 | Cspg2 | 5.05 |
| 621246 | interferon concensus sequence binding protein | Icsbp | 4.91 |
| 427469 | ethanol induced gene product EIG180 |  | 4.85 |
| 440344* | glutamine synthetase | Glns | 4.79 |
| 657528* | CREBBP/EP300 inhibitory protein 1 |  | 4.79 |
| 477860 | T-box 2 | Tbx2 | 4.66 |
| 737803* | histocompatibility 2, class II antigen E beta | H2-Eb1 | 4.63 |
| 671845 | platelet derived growth factor, alpha | Pdgfa | 4.56 |
| 426965 | crystallin, alpha C | Cryac | 4.55 |
| 1245128 | uroplakin 3 | Upk3 | 4.54 |
| 671834 | cadherin 2 | Cdh2 | 4.39 |
| 949472 | tumor necrosis factor, alpha-induced protein 2 | Tnfaip2 | 4.19 |
| 597336 | Zinc finger protein 275 | Zfp275 | 4.06 |
| 680894 | glial cell line derived neurotrophic factor family receptor alpha 1 | Gfra1 | 4.05 |
| 721409 | X-linked lymphocyte-regulated 4 | Xlr4 | 4.04 |
| 402348 | calponin 2 | Cnn2 | 4 |
| 329780 | cadherin 3 | Cdh3 | 3.96 |
| 427016 | paternally expressed 3 | Peg3 | 3.92 |
| 481883 | sialyltransferase 1 (beta-galactoside alpha-2,6-sialyltransferase) | Siat1 | 3.92 |
| 851569 | guanine nucleotide binding protein, alpha inhibiting 1 | Gnai1 | 3.88 |
| 1247541 | apolipoprotein D | Apod | 3.86 |
| 443870 | keratin complex 2, basic, gene 7 |  | 3.84 |
| 747104* | glypican 3 | Gpc3 | 3.81 |
| 482170 | four jointed box 1 (Drosophila) | Fjx1 | 3.78 |
| 373716 | tumor-associated calcium signal transducer 2 | Tacstd2 | 3.69 |
| 331186 | caveolin, caveolae protein, 22 kDa | Cav | 3.67 |
| 438506 | erythrocyte protein band 4.1-like 3 | Epb4.1l3 | 3.66 |
| 423942 | wingless-related MMTV integration site 5B | Wnt5b | 3.66 |
| 657264 | glypican 3 | Gpc3 | 3.66 |
| 482943 | odd-skipped related 1 (Drosophila) | Osr1 | 3.65 |
| 695667* | cytochrome P450, 2f2 | Cyp2f2 | 3.64 |
| 678765 | extracellular matrix protein 1 | Ecm1 | 3.61 |
| 596348 | aldehyde dehydrogenase family 1, subfamily A7 | Aldh1a4 | 3.46 |
| 717226 | lectin, galactose binding, soluble 3 | Lgals3 | 3.44 |
| 935316 | E26 avian leukemia oncogene 2, 3' domain | Ets2 | 3.44 |
| 419684 | mesenchyme homeobox 1 | Meox1 | 3.39 |
| 477177 | Ena-vasodilator stimulated phosphoprotein | Evl | 3.29 |
| 536306 | procollagen, type I, alpha 1 | Col1a1 | 3.27 |
| 1247224 | cathepsin E | Ctse | 3.25 |
| 821060 | growth arrest and DNA-damage-inducible 45 gamma | Gadd45g | 3.2 |
| 316409 | thymus cell antigen 1, theta | Thy1 | 3.18 |
| 481146* | Kinesin heavy chain member 4 | Kif4 | 3.18 |
| 352938* | tumor-associated calcium signal transducer 2 | Tacstd2 | 3.15 |
| 598827 | aryl-hydrocarbon receptor | Ahr | 3.14 |
| 479367 | twist gene homolog, (Drosophila) | Twist | 3.13 |
| 671910 | follistatin-like | Fstl | 3.1 |
| 427319 | ephrin B1 | Efnb1 | 3.07 |
| 736372* | tenascin C | Tnc | 3.07 |
| 1244332 | naked cuticle 1 homolog (Drosophila) | Nkd2 | 3.06 |
| 596968 | caveolin, caveolae protein, 22 kDa | Cav | 3.05 |
| 659857 | B-cell lymphoma/leukaemia 11B | Bcl11b | 3.02 |
| 475631 | calcium channel, voltage-dependent, T type, alpha 1G subunit | Cacna1g | 3.01 |
| 478472 | RAS p21 protein activator 3 | Rasa3 | 3.01 |
| 948509 | caveolin, caveolae protein, 22 kDa | Cav | 3 |
| 457038 | semaF cytoplasmic domain associated protein 2 | Semcap2-pending | 2.99 |
| 902923 | tissue inhibitor of metalloproteinase 2 | Timp2 | 2.98 |
| 1246411 | lymphocyte antigen 6 complex, locus A | Ly6 | 2.93 |
| 790532 | cell death-inducing DNA fragmentation factor, alpha subunit-like effector B | Cideb | 2.88 |
| 352450 | procollagen, type VI, alpha 1 | Col6a1 | 2.86 |
| 717191 | hypothetical protein LOC270138 | Ets1 | 2.86 |
| 749361 | chemokine (C-C) receptor 2 | Cmkbr2 | 2.84 |
| 903046 | transcription factor 4 | Tcf4 | 2.83 |
| 573301 | CD97 antigen | Cd97 | 2.82 |
| 580715 | dihydropyrimidinase-like 3 | Ly6 | 2.82 |
| 402738 | matrix metalloproteinase 2 | Mmp2 | 2.8 |
| 1054367 | myosin heavy chain 11, smooth muscle | Myh11 | 2.78 |
| 831635 | cholecystokinin | Cck | 2.77 |
| 891389 | pleckstrin homology-like domain, family A, member 3 | Phlda3 | 2.77 |
| 481469 | Mrps18b | 2400002C15Rik | 2.76 |
| 1245874 | solute carrier family 2 (facilitated glucose transporter), member 5 | Slc2a5 | 2.76 |
| 387280* | adenylate cyclase 7 | Adcy7 | 2.75 |
| 386559 | Bcl-2-related ovarian killer protein | Bok | 2.74 |
| 890598 | fatty acid binding protein 5, epidermal | Fabp5 | 2.74 |
| 467313 | cadherin 2 | Cdh2 | 2.73 |
| 420322 | procollagen, type III, alpha 1 | Col3a1 | 2.71 |
| 476784 | LIM only 1 | Lmo1 | 2.7 |
| 678523 | hemolytic complement | Hc | 2.68 |
| 803251 | ribonuclease, RNase A family 4 | Rnase4 | 2.68 |
| 331264 | twist gene homolog, (Drosophila) | Twist | 2.67 |
| 618001 | serine/threonine kinase 10 | Stk10 | 2.67 |
| 803897 | B-box and SPRY domain containing |  | 2.65 |
| 850971 | split hand/foot deleted gene 1 | Shfdg1 | 2.65 |
| 533117* | histidine triad nucleotide binding protein | Hint | 2.65 |
| 863587 | EH-domain containing 3 | Ehd3 | 2.64 |
| 618271 | histocompatibility 2, class II antigen A, beta 1 | H2-Ab1 | 2.63 |
| 465986 | DEAD/H (Asp-Glu-Ala-Asp/His) box polypeptide 26 | Ddx26 | 2.6 |
| 401838 | laminin, gamma 1 | Lamc1 | 2.59 |
| 672201 | crystallin, alpha B | Cryab | 2.56 |
| 737998 | carboxylesterase 2 |  | 2.55 |
| 695687* | calponin 2 | Cnn2 | 2.54 |
| 1196127 | stanniocalcin 2 | Stc2 | 2.53 |
| 367435 | biglycan | Bgn | 2.52 |
| 317268 | protease, serine, 20 | Prss20-pending | 2.51 |
| 423915 | development and differentiation enhancing | Ddef1 | 2.51 |
| 445075 | procollagen, type V, alpha 2 | Col5a2 | 2.51 |
| 656375 | FK506 binding protein 7 (23 kDa) | Fkbp7 | 2.51 |
| 862840 | interferon concensus sequence binding protein | Icsbp | 2.51 |
| 348037 | fibrinogen, B beta polypeptide | Fgb | 2.5 |
| 437699 | glucocorticoid-induced gene 1 |  | 2.5 |
| 747136* | high mobility group box 2 | Hmgb2 | 2.5 |
| 920268* | high mobility group AT-hook 1 | Hmga1 | 2.49 |
| 1246780 | RAS-related C3 botulinum substrate 2 | Rac2 | 2.49 |
| 467107 | procollagen, type V, alpha 2 | Col5a2 | 2.48 |
| 476264 | RIKEN cDNA 1110036H20 gene | AW215636 | 2.48 |
| 597280 | procollagen, type V, alpha 2 | Col5a2 | 2.48 |
| 367627 | glutathione S-transferase, alpha 4 | Gsta4 | 2.47 |
| 761622 | Tnfa-induced adipose-related protein | Tiarp-pending | 2.46 |
| 765332 | vinculin | Vcl | 2.46 |
| 427360 | filamin-like protein |  | 2.44 |
| 575397 | chemokine (C-C motif) ligand 6 | Scya6 | 2.44 |
| 492502 | bone morphogenetic protein 1 | Bmp1 | 2.43 |
| 1247588 | adipocyte complement related protein of 30 kDa | Acrp30 | 2.41 |
| 317218 | FK506 binding protein 9 | Fkbp9 | 2.4 |
| 1313559 | mucin 1, transmembrane | Muc1 | 2.4 |
| 440563 | platelet factor 4 | Pf4 | 2.34 |
| 443884 | tumor necrosis factor, alpha-induced protein 2 | Tnfaip2 | 2.33 |
| 890932 | calcium channel, voltage-dependent, alpha2/delta subunit 1 | Cacna2d1 | 2.33 |
| 1196394 | myristoylated alanine rich protein kinase C substrate | Macs | 2.33 |
| 1244224 | proteinase 3 | Prtn3 | 2.33 |
| 1347203 | prostaglandin E receptor 2 (subtype EP2) | Ptger2 | 2.33 |
| 425855 | lymphocyte antigen 6 complex, locus C | Ly6c | 2.31 |
| 693146 | glutamine synthetase | Glns | 2.3 |
| 1383141 | RIKEN cDNA 5730591C18 gene | Tcea1 | 2.3 |
| 424617 | angiotensin receptor-like 1 | Agtrl1 | 2.29 |
| 472801 | microfibrillar associated protein 5 | Mfap5-pending | 2.29 |
| 747038 | procollagen, type III, alpha 1 | Col3a1 | 2.29 |
| 1265616 | angio-associated migratory protein | Aamp-rs | 2.29 |
| 374228 | fibromodulin | Fmod | 2.28 |
| 440564 | E26 avian leukemia oncogene 2, 3' domain | Ets2 | 2.28 |
| 622288 | interleukin 1 receptor, type I | Il1r1 | 2.28 |
| 1195716 | tweety homolog 2 (Drosophila) | Ttyh2 | 2.28 |
| 478504 | calpain 6 | Capn6 | 2.27 |
| 573265* | annexin A3 | Anxa3 | 2.27 |
| 1314183 | vinculin | Vcl | 2.27 |
| 762558 | myristoylated alanine rich protein kinase C substrate | Macs | 2.26 |
| 484299 | endoglin | Eng | 2.25 |
| 403597 | bone morphogenetic protein 1 | Bmp1 | 2.24 |
| 481837 | laminin, gamma 1 | Lamc1 | 2.24 |
| 581007 | solute carrier family 13 (sodium-dependent dicarboxylate transporter), member 3 | Slc13a3 | 2.24 |
| 680198 | ATP-binding cassette, sub-family G (WHITE), member 5 | Abcg5 | 2.23 |
| 1449094 | proteinase 3 | Prtn3 | 2.22 |
| 1246028 | T-cell receptor alpha chain | Tcra | 2.22 |
| 577821 | thyroid hormone receptor-associated protein 100 kDa | Trap100-pending | 2.21 |
| 723118 | B-cell CLL/lymphoma 11A (zinc finger protein) | Bcl11a | 2.2 |
| 474298 | stanniocalcin | Stc | 2.19 |
| 352302 | septin 9 | 9-Sep | 2.18 |
| 355112 | ral guanine nucleotide dissociation stimulator | Rgds | 2.17 |
| 1245706 | sialyltransferase 1 (beta-galactoside alpha-2,6-sialyltransferase) | Siat1 | 2.17 |
| 480467 | aquaporin 1 | Aqp1 | 2.16 |
| 617200 | myristoylated alanine rich protein kinase C substrate | Macs | 2.15 |
| 618681 | myristoylated alanine rich protein kinase C substrate | Macs | 2.15 |
| 440755* | Makorin, ring finger protein, 3 | Mkrn3 | 2.14 |
| 1510567 | serum amyloid A 3 | Saa3 | 2.14 |
| 949520 | frizzled homolog 7 (Drosophila) | Fzd7 | 2.13 |
| 493702 | DNA segment, Chr 8, ERATO Doi 91, expressed | D8Ertd91e | 2.12 |
| 482641 | RNA, U22 small nucleolar | Rnu22 | 2.12 |
| 637568 | Fas death domain-associated protein | Daxx | 2.12 |
| 480620 | procollagen, type VI, alpha 3 | Col6a3 | 2.11 |
| 638791 | mannosidase 1, alpha | Man1a | 2.11 |
| 720554 | DEAD/H (Asp-Glu-Ala-Asp/His) box polypeptide 21 (RNA helicase II/Gu) | Ddx21 | 2.11 |
| 891131 | eosinophil-associated ribonuclease 2 | Ear2 | 2.11 |
| 401608 | AXL receptor tyrosine kinase | Axl | 2.1 |
| 634581 | F-box only protein 25 | 9130015I06Rik | 2.1 |
| 746939 | gap junction membrane channel protein alpha 4 | Gja4 | 2.1 |
| 752429 | LIM only 2 | Lmo2 | 2.09 |
| 777640 | complement component factor h | Cfh | 2.09 |
| 874740 | guanine nucleotide binding protein, alpha inhibiting 1 | Gnai1 | 2.09 |
| 751710 | neuroepithelial cell transforming gene 1 | Net1 | 2.08 |
| 406947 | peptidylprolyl isomerase C | Ppic | 2.07 |
| 734101* | transforming growth factor, beta induced, 68 kDa | Tgfbi | 2.07 |
| 851060 | septin 1 | 1-Sep | 2.07 |
| 437674 | high mobility group 20 B | Hmg20b | 2.06 |
| 1247905 | chemokine (C-X-C motif) ligand 9 | Scyb9 | 2.06 |
| 963071 | whn-dependent transcript 2 | Wdt2 | 2.06 |
| 401327 | keratin complex 2, basic, gene 4 | Krt2-4 | 2.05 |
| 439737 | Notch-regulated ankyrin repeat protein | Nrarp | 2.05 |
| 1348835 | ubiquitin specific protease 11 |  | 2.03 |
| 597832 | CUG triplet repeat,RNA binding protein 2 | Cugbp2 | 2.02 |
| 597352* | thymopoietin | Tmpo | 2.02 |
| 1382148 | procollagen, type V, alpha 2 | Col5a2 | 2.02 |
| 1400335 | myristoylated alanine rich protein kinase C substrate | Macs | 2.02 |
| 948896* | forkhead box P1 | Foxp1 | 2.01 |
| 403071 | osteoblast specific factor 2 (fasciclin I-like) | Osf2-pending | 2 |
| 850420 | ATP-binding cassette, sub-family A (ABC1), member 2 | Abca2 | 2 |
| 750503 | ELK3, member of ETS oncogene family | Elk3 | 2 |
| 697191 | thymopoietin | Tmpo | 1.99 |
| 355445 | nucleoporin 210 | Pom210 | 1.98 |
| 482995 | transforming growth factor beta 1 induced transcript 4 | Tgfb1i4 | 1.98 |
| 1314351 | ataxin-1 ubiquitin-like interacting protein | Ubin-pending | 1.98 |
| 477541 | acupuncture induced gene 1 | Aig1-pending | 1.97 |
| 572428 | cyclin D1 | Ccnd1 | 1.97 |
| 875105 | cell division cycle 37 homolog (S. cerevisiae)-like | 2700033A15Rik | 1.97 |
| 406897* | retinol binding protein 1, cellular | Rbp1 | 1.96 |
| 441346* | myelocytomatosis oncogene | Myc | 1.96 |
| 463860 | platelet-derived growth factor, C polypeptide | Pdgfc | 1.96 |
| 820833 | Insulin-like growth factor binding protein 7 | Igfbp7 | 1.96 |
| 1054137 | PTPRF interacting protein, binding protein 1 (liprin beta 1) | 4632409B19Rik | 1.96 |
| 466295 | ribosomal protein S15 | Rps15 | 1.95 |
| 620101 | double cortin and calcium/calmodulin-dependent protein kinase-like 1 | Dcamkl1 | 1.95 |
| 737898 | carnitine palmitoyltransferase 1, liver | Cpt1a | 1.95 |
| 620221 | CUG triplet repeat,RNA binding protein 2 | Cugbp2 | 1.93 |
| 864344 | monocyte to macrophage differentiation-associated | Mmd | 1.93 |
| 418453 | Sialyltranferase 5 | Siat5 | 1.92 |
| 438774 | CCR4 carbon catabolite repression 4-like (S. cerevisiae) | Ccr4 | 1.92 |
| 597005* | fibronectin 1 | Fn1 | 1.92 |
| 746644 | lumican | Lum | 1.92 |
| 355035 | histone deacetylase 2 | Hdac2 | 1.91 |
| 698181 | elastin | Eln | 1.91 |
| 426524* | Eye absent 2 homolog (Drosophila) | Eya2 | 1.9 |
| 1179115 | transcription factor E2a | Tcfe2a | 1.9 |
| 875395* | 5'-3' exoribonuclease 2 | Xrn2 | 1.89 |
| 692609 | protein C | Proc | 1.89 |
| 532350 | integral membrane protein 2A | Itm2 | 1.89 |
| 1247470 | Tnfa-induced adipose-related protein | Tiarp-pending | 1.89 |
| 332687 | cDNA sequence BC002262 | BC002262 | 1.88 |
| 418576 | nucleoporin 210 | Pom210 | 1.88 |
| 576245 | ATP-binding cassette, sub-family C (CFTR/MRP), member 5 | Abcc5a | 1.88 |
| 680450 | epithelial protein lost in neoplasm | Eplin-pending | 1.88 |
| 760769 | epithelial protein lost in neoplasm | Eplin-pending | 1.87 |
| 692257 | matrix metalloproteinase 23 | Mmp23 | 1.87 |
| 385581 | high mobility group nucleosomal binding domain 3 | Hmgn3 | 1.86 |
| 409069 | transformation related protein 53 | Trp53 | 1.86 |
| 423147 | tweety homolog 2 (Drosophila) | Ttyh2 | 1.86 |
| 876390 | mannose receptor, C type 1 | Mrc1 | 1.86 |
| 637493* | Cerebellar ataxia 3 | Cla3 | 1.85 |
| 949055 | E26 avian leukemia oncogene 2, 3' domain | Ets2 | 1.85 |
| 426976 | branched chain aminotransferase 1, cytosolic | Bcat1 | 1.84 |
| 439383* | slit homolog 3 (Drosophila) | Slit3 | 1.84 |
| 698175 | nidogen 1 | Nid1 | 1.84 |
| 315993 | laminin, gamma 2 | Lamc2 | 1.82 |
| 1195358 | WNT1 inducible signaling pathway protein 2 | Wisp2 | 1.82 |
| 1230769 | carboxypeptidase X 1 (M14 family) | CPX-1 | 1.82 |
| 573784 | phosphoserine aminotransferase | D8Ertd814e | 1.81 |
| 697416 | placenta-specific 8 | D5Wsu111e | 1.8 |
| 1396418 | carbonic anhydrase 3 | Car3 | 1.79 |
| 734861 | S-phase kinase-associated protein 2 (p45) | Skp2 | 1.77 |
| 418633 | macrophage galactose N-acetyl-galactosamine specific lectin 1 | Mgl | 1.75 |
| 419146 | cyclin-dependent kinase inhibitor 1A (P21) | Cdkn1a | 1.74 |
| 574375 | RAS p21 protein activator 2 | Rasa2 | 1.72 |
| 864664 | transmembrane protein 2 | Tmem2 | 1.72 |
| 723554 | heterogeneous nuclear ribonucleoprotein D-like | Hnrpdl | 1.7 |
| 1313586 | LPS-responsive beige-like anchor | Lrba | 1.69 |
| 439102 | thrombospondin 2 | Thbs2 | 1.61 |
| 465403 | breast cancer anti-estrogen resistance 3 | Bcar3 | 1.52 |
| 571816* | DnaJ (Hsp40) homolog, subfamily B, member 12 | Dnajb12 | 1.52 |
| 634625 | runt related transcription factor 1 | Runx1 | 1.5 |
| 720207 | leucine-rich alpha-2-glycoprotein | Lrg-pending | 1.47 |
| 746853 | phosphoserine aminotransferase | D8Ertd814e | 1.46 |
| 1179683 | chloride channel calcium activated 1 |  | 1.24 |
| 736854 | carboxypeptidase X 1 (M14 family) | CPX-1 | 1.03 |
| 463388 | BCL2/adenovirus E1B 19 kDa-interacting protein 1, NIP3 | Bnip3 | 0.62 |
| 778942 | activating transcription factor 2 | Atf2 | 0.59 |
| 635299 | hemogen | Hgn-pending | 0.59 |
| 765935* | cholinergic receptor, nicotinic, beta polypeptide 1 (muscle) | Chrnb1 | 0.59 |
| 1382817 | membrane-associated protein 17 | Map17-pending | 0.59 |
| 1247073 | fibroblast growth factor receptor-like 1 | Fgfrl1 | 0.59 |
| 777238 | ELL-related RNA polymerase II, elongation factor |  | 0.58 |
| 850251 | syntaxin 8 | Stx8 | 0.57 |
| 388244 | TEA domain family member 2 | Tead2 | 0.56 |
| 478354 | beta-spectrin 3 | Spnb3 | 0.56 |
| 920235 | RAB25, member RAS oncogene family | Rab25 | 0.56 |
| 697092 | phosphatidic acid phosphatase type 2c | Ppap2c | 0.56 |
| 1245198 | Notch gene homolog 1, (Drosophila) | Notch1 | 0.56 |
| 1195103 | FXYD domain-containing ion transport regulator 3 | Fxyd3 | 0.56 |
| 334876 | pyruvate dehydrogenase kinase, isoenzyme 4 | Pdk4 | 0.55 |
| 580579 | rabaptin 5 | Rab5ep-pending | 0.55 |
| 699181 | solute carrier family 25 (mitochondrial carnitine/acylcarnitine translocase), member 20 | Slc25a20 | 0.55 |
| 864769 | procollagen, type V, alpha 3 | Col5a3 | 0.55 |
| 1230847 | galactose-4-epimerase, UDP | Gale | 0.55 |
| 1195754 | mitogen-activated protein kinase 11 | Mapk11 | 0.55 |
| 1247350 | Cbp/p300-interacting transactivator, with Glu/Asp-rich carboxy-terminal domain, 4 | Cited4 | 0.55 |
| 963913 | cytochrome b-561 | Cyb561 | 0.55 |
| 944891 | ELL-related RNA polymerase II, elongation factor |  | 0.54 |
| 401253 | plexin A3 | Plxn3 | 0.54 |
| 891229 | DnaJ (Hsp40) homolog, subfamily A, member 4 | Dnaja4 | 0.54 |
| 314979 | lysosomal apyrase-like 1 | 1200014F22Rik | 0.53 |
| 642372 | glycoprotein 9 (platelet) | Gp9 | 0.53 |
| 876698* | annexin A6 | Anxa6 | 0.53 |
| 850199 | reticulon 3 | Rtn3 | 0.53 |
| 1069274 | hypothetical protein MGC28888 | D5Wsu46e | 0.53 |
| 1248322 | Isovaleryl coenzyme A dehydrogenase | Ivd | 0.53 |
| 441645 | LIM domains containing 1 | Limd1 | 0.52 |
| 571367 | BCL2/adenovirus E1B 19 kDa-interacting protein 1, NIP3 | Bnip3 | 0.52 |
| 535763 | ubiquitin conjugating enzyme 7 interacting protein 3 | Ubce7ip3-pending | 0.52 |
| 671280 | AT motif binding factor 1 | Atbf1 | 0.52 |
| 851317 | hypothetical protein MGC11687 |  | 0.52 |
| 1248009 | DNA segment, Chr 3, University of California at Los Angeles 1 | D3Ucla1 | 0.52 |
| 350180* | thioredoxin interacting protein | Vdup1-pending | 0.51 |
| 419516 | transient receptor potential cation channel, subfamily M, member 1 | Trpm1 | 0.51 |
| 407116* | interferon dependent positive acting transcription factor 3 gamma | Isgf3g | 0.51 |
| 426304 | 7-dehydrocholesterol reductase | Dhcr7 | 0.51 |
| 480896 | basic helix-loop-helix domain containing, class B2 | Bhlhb2 | 0.51 |
| 717787 | ras-like protein | Pigf | 0.51 |
| 748015* | immunity-associated protein, 38 kDa | Imap38 | 0.51 |
| 1178551 | DNA segment, Chr 14, ERATO Doi 226, expressed | D14Ertd226e | 0.51 |
| 1245704 | inhibitor of kappaB kinase epsilon | Ikbke | 0.51 |
| 1397295 | hypothetical protein LOC232227 | D6Ertd349e | 0.51 |
| 483636 | vascular Rab-GAP/TBC-containing | Vrp-pending | 0.5 |
| 580142 | fructosamine 3 kinase | Fn3k | 0.5 |
| 699236 | protein phosphatase 3, catalytic subunit, alpha isoform | Ppp3ca | 0.5 |
| 718521 | rabaptin 5 | Rab5ep-pending | 0.5 |
| 762542 | ras-like protein | Pigf | 0.5 |
| 1346722 | ELL-related RNA polymerase II, elongation factor |  | 0.5 |
| 1398634 | small inducible cytokine subfamily A17 | Scya17 | 0.5 |
| 1448588 | DNA segment, Chr 8, ERATO Doi 790, expressed | D8Ertd790e | 0.5 |
| 760889 | galactose-4-epimerase, UDP | Gale | 0.49 |
| 333636* | excision repair cross-complementing rodent repair deficiency, complementation group 2 | Ercc2 | 0.49 |
| 385528 | similar to choline transporter-like protein |  | 0.49 |
| 437213 | thrombospondin 4 | Thbs4 | 0.49 |
| 571369* | Tripartite motif protein 8 | Trim8 | 0.49 |
| 583808 | guanylate nucleotide binding protein 2 | Gbp2 | 0.49 |
| 873928 | xanthine dehydrogenase | Xdh | 0.49 |
| 920425 | histocompatibility 2, L region | H2-L | 0.49 |
| 681031 | hypothetical protein MGC27648 |  | 0.49 |
| 1245764 | vasoactive intestinal peptide receptor 1 | Vipr1 | 0.49 |
| 459170 | Eph receptor A2 | Epha2 | 0.48 |
| 483763 | thrombospondin 3 | Thbs3 | 0.48 |
| 576775 | transporter 1, ATP-binding cassette, sub-family B (MDR/TAP) | Abcb2 | 0.48 |
| 583657 | Nedd4 WW binding protein 4 | N4wbp4-pending | 0.48 |
| 643755 | nucleoporin 88kDa | Prei2 | 0.48 |
| 777549 | protein phosphatase 3, catalytic subunit, alpha isoform | Ppp3ca | 0.48 |
| 876313 | similar to Mid-1-related chloride channel 1 |  | 0.48 |
| 1398166 | deleted in polyposis 1 | Dp1 | 0.48 |
| 480575 | branched chain ketoacid dehydrogenase kinase | Bckdk | 0.47 |
| 620546 | protein phosphatase 3, catalytic subunit, alpha isoform | Ppp3ca | 0.47 |
| 693565* | carnitine O-octanoyltransferase | Crot | 0.47 |
| 1067326 | ras-like protein | Pigf | 0.47 |
| 1246264 | adipsin | Adn | 0.47 |
| 407096* | Decay accelerating factor 1 | Daf1 | 0.46 |
| 425562 | diacylglycerol kinase zeta |  | 0.46 |
| 443865 | similar to DKFZP564O0823 protein |  | 0.46 |
| 456862 | sorting nexin 1 | Snx1 | 0.46 |
| 598214 | PTK2 protein tyrosine kinase 2 beta | Ptk2b | 0.46 |
| 653513 | Tangerine | LOC114601 | 0.46 |
| 403493 | oxysterol binding protein-like 1A | AB017026 | 0.45 |
| 420487 | calcium channel, voltage-dependent, beta 3 subunit | Cacnb3 | 0.45 |
| 579998* | acetyl-Coenzyme A synthetase 2 (AMP forming)-like | AI788978 | 0.45 |
| 599164 | thymidylate kinase family LPS-inducible member | Tyki | 0.45 |
| 1248298 | absent in melanoma 1 | AI463325 | 0.45 |
| 1384121 | mitchondrial ribosomal protein S7 | Mrps7 | 0.45 |
| 1397318 | guanine nucleotide binding protein, beta 1 | Gnb1 | 0.45 |
| 475944 | ectonucleotide pyrophosphatase/phosphodiesterase 5 | Enpp5 | 0.44 |
| 579349 | epoxide hydrolase 2, cytoplasmic | Ephx2 | 0.44 |
| 618165 | non-catalytic region of tyrosine kinase adaptor protein 1 | Nck1 | 0.44 |
| 850441 | syndecan 4 | Sdc4 | 0.44 |
| 876221 | dual specificity phosphatase 12 | Dusp12 | 0.44 |
| 1248586 | G7e protein | G7e-pending | 0.44 |
| 1314857 | angiopoietin-like 2 | Angptl2 | 0.44 |
| 1068129 | papilin, proteoglycan-like sulfated glycoprotein |  | 0.44 |
| 441695 | claudin 3 | Cldn3 | 0.43 |
| 466624 | fibrosin | Trim8 | 0.43 |
| 493675 | actinin alpha 3 | Actn3 | 0.43 |
| 493182 | solute carrier family 27 (fatty acid transporter), member 4 | Slc27a4 | 0.43 |
| 523123* | ectonucleotide pyrophosphatase/phosphodiesterase 5 | Enpp5 | 0.43 |
| 1511643 | CD36 antigen | Cd36 | 0.43 |
| 1054558 | electron transferring flavoprotein, alpha polypeptide | D9Ertd394e | 0.43 |
| 463941 | vesicular inhibitory amino acid transporter | Viaat | 0.42 |
| 472850 | MIC2 (monoclonal Imperial Cancer Research Fund 2)-like 1 | Mtmr1 | 0.42 |
| 478428 | old astrocyte specifically induced substance | Oasis-pending | 0.42 |
| 680964* | matrix metalloproteinase 15 | Mmp15 | 0.42 |
| 1448721 | aquaporin 5 | Aqp5 | 0.42 |
| 1247913 | old astrocyte specifically induced substance | Oasis-pending | 0.42 |
| 479750* | adenylate kinase 3 alpha-like | Akl3l-pending | 0.41 |
| 620057 | thymidylate kinase family LPS-inducible member | Tyki | 0.41 |
| 776890 | argininosuccinate synthetase 1 | Ass1 | 0.41 |
| 764542* | epoxide hydrolase 2, cytoplasmic | Ephx2 | 0.41 |
| 1196011 | insulin-like growth factor binding protein 5 | Igfbp5 | 0.41 |
| 1381870 | vav 3 oncogene | Vav3 | 0.41 |
| 314850 | protein tyrosine phosphatase, receptor-type, F interacting protein, binding protein 2 | Ppfibp2 | 0.4 |
| 386236* | a disintegrin and metalloproteinase domain 12 (meltrin alpha) | Adam12 | 0.4 |
| 477962 | histone 1, H1c | H1f2 | 0.4 |
| 737041* | betaine-homocysteine methyltransferase 2 | Bhmt2 | 0.4 |
| 775893* | CD 81 antigen | Cd81 | 0.4 |
| 851686 | fibroblast growth factor receptor-like 1 | Fgfrl1 | 0.4 |
| 935387 | solute carrier family 31, member 2 | Slc31a2 | 0.4 |
| 334182* | amyotrophic lateral sclerosis 2 (juvenile) homolog (human) | Als2 | 0.39 |
| 427197 | solute carrier family 16 (monocarboxylic acid transporters), member 2 | Slc16a2 | 0.39 |
| 444023 | delta-like 1 (Drosophila) | Dll1 | 0.39 |
| 464200 | insulin-like growth factor 2 receptor | Igf2r | 0.39 |
| 949722 | B lymphocyte gene 1 | Bce1-pending | 0.39 |
| 920069 | cyclin-dependent kinase 9 (CDC2-related kinase) | Cdk9 | 0.39 |
| 779711 | cyclin-dependent kinase-like 2 (CDC2-related kinase) | Cdkl2 | 0.38 |
| 314215 | tyrosine aminotransferase | Tat | 0.38 |
| 717926 | ubiquitin specific protease 18 | Usp18 | 0.38 |
| 831918* | UDP-Gal:betaGlcNAc beta 1,4-galactosyltransferase, polypeptide 6 | B4galt6 | 0.38 |
| 849849 | protein tyrosine phosphatase, non-receptor type 4 | Ptpn4 | 0.38 |
| 427314* | cartilage derived retinoic acid sensitive protein | Cdrap | 0.37 |
| 870852* | pantophysin | Pphn | 0.37 |
| 693148 | MIC2 (monoclonal Imperial Cancer Research Fund 2)-like 1 | Mic2l1 | 0.37 |
| 671661 | insulin-like growth factor binding protein 5 | Igfbp5 | 0.37 |
| 719691 | fetuin beta | Fetub | 0.37 |
| 948786 | proteosome (prosome, macropain) subunit, beta type 9 (large multifunctional protease 2) | Psmb9 | 0.37 |
| 390236 | Sel1 (suppressor of lin-12) 1 homolog (C. elegans) | Sel1h | 0.36 |
| 480073 | guanine nucleotide binding protein, alpha o | Gnao | 0.36 |
| 481381 | glutamate oxaloacetate transaminase 1, soluble | Got1 | 0.36 |
| 575698 | 2'-5' oligoadenylate synthetase-like 2 | Oasl2 | 0.36 |
| 762313 | phosphodiesterase 9A | Pde9a | 0.36 |
| 831701 | transcription factor 1 | Tcf1 | 0.36 |
| 890493 | adipose differentiation related protein | Adfp | 0.36 |
| 891119 | phosphofructokinase, liver, B-type | Pfkl | 0.36 |
| 595848* | immunoglobulin joining chain | Igj | 0.35 |
| 595950* | Rac/Cdc42 guanine nucleotide exchange factor (GEF) 6 | Arhgef6 | 0.35 |
| 872869 | S100 calcium binding protein A1 | S100a1 | 0.35 |
| 876463* | plasma glutamate carboxypeptidase | Pgcp-pending | 0.35 |
| 692546* | acyl-Coenzyme A dehydrogenase, short/branched chain | Acadsb | 0.35 |
| 733601 | transmembrane 4 superfamily member 1 | Tm4sf1 | 0.35 |
| 748275 | sialyltransferase 9 (CMP-NeuAc:lactosylceramide alpha-2,3-sialyltransferase) | Siat9 | 0.35 |
| 777513 | salvador homolog 1 (Drosophila) | Sav1 | 0.35 |
| 446084 | very low density lipoprotein receptor | Vldlr | 0.34 |
| 479269 | inositol 1,4,5-triphosphate receptor 3 | Itpr3 | 0.34 |
| 1383625 | syntrophin, basic 1 | Sntb1 | 0.34 |
| 1400768 | expressed sequence AA959686 | Rab18 | 0.34 |
| 481721 | inositol 1,4,5-triphosphate receptor 3 | Itpr3 | 0.33 |
| 658863 | general control of amino acid synthesis-like 2 (yeast) | Gcn5l2 | 0.33 |
| 692913 | carbonic anhydrase-like sequence 1 | Cals1 | 0.33 |
| 831668 | retinol binding protein 7, cellular | Rbp7 | 0.33 |
| 1067312 | general control of amino acid synthesis-like 2 (yeast) | Gcn5l2 | 0.33 |
| 1248560 | pantophysin | Pphn | 0.33 |
| 660287 | sialyltransferase 7 ((alpha-N-acetylneuraminyl 2,3-betagalactosyl-1,3)-N-acetyl galactosaminide alpha-2,6-sialyltransferase) D | Siat7d | 0.32 |
| 718716* | hemoglobin Z, beta-like embryonic chain | Hbb-bh1 | 0.32 |
| 735576 | RAB3B, member RAS oncogene family | Rab3b | 0.32 |
| 803337* | peroxisomal membrane protein 4 | Pmp24-pending | 0.32 |
| 733060 | actinin alpha 4 | Actn4 | 0.32 |
| 851605 | schlafen 2 | Slfn2 | 0.32 |
| 1178689 | chitinase 3-like 1 | Chi3l1 | 0.32 |
| 418952 | insulin-like growth factor binding protein 5 | Igfbp5 | 0.31 |
| 832113 | adipose differentiation related protein | Adfp | 0.31 |
| 439763 | ATP-binding cassette, sub-family G (WHITE), member 2 | Abcg2 | 0.3 |
| 466122* | solute carrier family 12, member 2 | Slc12a2 | 0.3 |
| 479786 | general control of amino acid synthesis-like 2 (yeast) | Gcn5l2 | 0.3 |
| 636513 | polymerase (RNA) II (DNA directed) polypeptide I | Rpo2-4 | 0.3 |
| 889753* | phosphodiesterase 9A | Pde9a | 0.3 |
| 1248377 | hemoglobin Z, beta-like embryonic chain | Hbb-bh1 | 0.3 |
| 1397810 | toll-like receptor 5 | Tlr5 | 0.3 |
| 483758* | RAB3D, member RAS oncogene family | Rab3d | 0.29 |
| 572463* | potassium channel, subfamily K, member 5 | Kcnk5 | 0.29 |
| 737364 | polymeric immunoglobulin receptor | Pigr | 0.29 |
| 1244615 | regucalcin gene promotor region related protein | Rgpr-pending | 0.29 |
| 1230273 | aquaporin 7 | Aqp7 | 0.29 |
| 634233 | phospholipid scramblase 3 | Plscr3 | 0.28 |
| 733456* | gamma-glutamyl hydrolase | Ggh | 0.28 |
| 1248573 | general control of amino acid synthesis-like 2 (yeast) | Gcn5l2 | 0.28 |
| 1476887 | type 1 tumor necrosis factor receptor shedding aminopeptidase regulator | Arts1-pending | 0.28 |
| 423596* | actinin alpha 4 | Actn4 | 0.27 |
| 482883 | nucleobindin 2 | Nucb2 | 0.27 |
| 493658 | lipocalin 2 | Lcn2 | 0.27 |
| 832584 | Fc receptor, IgG, low affinity III | Fcgr3 | 0.26 |
| 1364090 | phosphatidylinositol 3 kinase, regulatory subunit, polypeptide 4, p150 | Pik3r4 | 0.26 |
| 335369* | arachidonate 12-lipoxygenase, 12R type | Alox12b | 0.25 |
| 572542* | beta-2 microglobulin | B2m | 0.25 |
| 596447* | histocompatibility 2, Q region locus 7 | H2-Q7 | 0.25 |
| 694651* | histocompatibility 2, L region | H2-L | 0.25 |
| 656701 | dimethylarginine dimethylaminohydrolase 1 | Ddah1 | 0.25 |
| 850936 | S100 protein, beta polypeptide, neural+C565 | S100b | 0.25 |
| 1347651 | Eph receptor A2 | Epha2 | 0.25 |
| 1348439 | solute carrier family 12, member 2 | Slc12a2 | 0.25 |
| 419420 | oligodendrocyte transcription factor 2 | Olig2 | 0.24 |
| 621555* | hemoglobin, beta adult minor chain | Hbb-b1 | 0.24 |
| 622914* | serine/threonine kinase 39, STE20/SPS1 homolog (yeast) | Spak-pending | 0.24 |
| 820849 | forkhead box I1 | Fkh10 | 0.24 |
| 330336* | histocompatibility 2, K region | H2-K | 0.23 |
| 820122 | insulin-like growth factor binding protein, acid labile subunit | Igfals | 0.23 |
| 1314637 | carboxypeptidase D | Cpd | 0.23 |
| 389177 | hexokinase 1 | Hk1 | 0.22 |
| 876145* | TGFB inducible early growth response | Tieg | 0.22 |
| 736616 | laminin, alpha 4 | Lama4 | 0.22 |
| 579391 | carbonic anhydrase 2 | Car2 | 0.21 |
| 903419 | aldolase 1, A isoform | Aldo1 | 0.21 |
| 747208 | interferon-inducible GTPase | Iigp-pending | 0.21 |
| 596863 | interferon-inducible GTPase | Iigp-pending | 0.2 |
| 832158 | extracellular proteinase inhibitor | Expi | 0.2 |
| 1398011 | guanine nucleotide binding protein (G protein), gamma 10 | Gng10 | 0.2 |
| 373942 | heparan sulfate (glucosamine) 3-O-sulfotransferase 1 | Hs3st1 | 0.19 |
| 481691 | serine/threonine kinase 39, STE20/SPS1 homolog (yeast) | Spak-pending | 0.19 |
| 1265649 | malic enzyme, supernatant | Mod1 | 0.19 |
| 761326 | sine oculis-related homeobox 3 homolog (Drosophila) | Six3 | 0.18 |
| 805549* | solute carrier family 29 (nucleoside transporters), member 1 | Slc29a1 | 0.18 |
| 1248291 | caspase 11 | Casp11 | 0.18 |
| 1313822 | ethanol induced 6 | Etohi6 | 0.18 |
| 400632 | solute carrier family 21 (prostaglandin transporter), member 2 | Slc21a2 | 0.17 |
| 876655* | EGL nine homolog 3 (C. elegans) | Egln3 | 0.17 |
| 764385 | interferon-inducible GTPase | Iigp-pending | 0.17 |
| 535199* | s17 protein | S17-pending | 0.17 |
| 419138* | hairy/enhancer-of-split related with YRPW motif 1 | Hey1 | 0.16 |
| 425427* | potassium intermediate/small conductance calcium-activated channel, subfamily N, member 4 | Kcnn4 | 0.16 |
| 441788* | Rap1, GTPase-activating protein 1 | Rap1ga1 | 0.16 |
| 481108 | cartilage oligomeric matrix protein | Comp | 0.15 |
| 618272* | caspase 11 | Casp11 | 0.15 |
| 680512* | solute carrier family 29 (nucleoside transporters), member 1 | Slc29a1 | 0.15 |
| 697010* | transcobalamin 2 | Tcn2 | 0.15 |
| 752301 | 2'-5' oligoadenylate synthetase 1A | Oas1a | 0.15 |
| 752233* | hairy/enhancer-of-split related with YRPW motif 1 | Hey1 | 0.15 |
| 1383463 | potassium intermediate/small conductance calcium-activated channel, subfamily N, member 4 | Kcnn4 | 0.15 |
| 441857 | homer homolog 2 (Drosophila) | Homer2-pending | 0.14 |
| 638204 | ethanol decreased 2 | Etohd2 | 0.14 |
| 775484 | plexin A3 | Plxn3 | 0.13 |
| 576815* | small inducible cytokine subfamily C, member 1 (lymphotactin) | Scyc1 | 0.13 |
| 738285 | lysine oxoglutarate reductase, saccharopine dehydrogenase | Lorsdh | 0.13 |
| 535602 | G protein beta subunit-like | Gbl-pending | 0.13 |
| 851207 | arsenate resistance protein 2 | Ars2-pending | 0.13 |
| 550766 | caspase 1 | Casp1 | 0.12 |
| 423028* | procollagen, type XI, alpha 1 | Col11a1 | 0.11 |
| 390313* | aldolase 3, C isoform | Aldo3 | 0.09 |
| 775253* | A kinase (PRKA) anchor protein (gravin) 12 | Akap12 | 0.08 |
| 481151* | procollagen, type IX, alpha 1 | Col9a1 | 0.08 |
| 864409* | CD36 antigen | Cd36 | 0.08 |
| 806940* | A kinase (PRKA) anchor protein (gravin) 12 | Akap12 | 0.08 |
| 875031 | casein kappa | Csnk | 0.08 |
| 634658 | vesicle-associated membrane protein 3 | Vamp3 | 0.07 |
| 479316* | transmembrane protein with EGF-like and two follistatin-like domains 2 | Tmeff2 | 0.06 |
| 402800* | nephronectin | Npnt | 0.04 |
| 697864 | peptidoglycan recognition protein | Pglyrp | 0.04 |
| 329940* | Carbonic anhydrase 6 | Car6 | 0.01 |

*Genes also differentially expressed among tumors from MMTV-Neu , MMTV-Ha-Ras, MMTV-c-Myc, MMTV-polyoma middle T antigen (PyMT), C3(1)/simian virus 40 (SV40) T/t antigen, and Wap-SV40 T/t antigen TG mice using the 8.7k chips reported by Desai *et al.* [7].

**Expression ratio is computed by dividing the average expression value of MMTV-Wnt-1-induced tumors by that of MMTV-Neu-induced tumors. P=<0.001. ESTs and riken cDNAs were excluded. Data generated from 15k chips.
