# Supplementary material for: Changes in gene expression during the development of mammary tumors in MMTV-Wnt-1 transgenic mice
Source: Genome Biol. 2005 Sep 30;6(10):R84. doi: 10.1186/gb-2005-6-10-r84 (PMC1257467; doi:10.1186/gb-2005-6-10-r84)
Supplement: Additional File 2 — A table listing genes differentially expressed between Ha-Ras mutant and Ha-Ras wild-type tumors in MMTV-Wnt-1 mice [file gb-2005-6-10-r84-S2.doc]

| **Additional data file 2. Genes differentially expressed between Ha-Ras mutant and Ha-Ras wild-type tumors in MMTV-Wnt-1 mice** | | | |
| --- | --- | --- | --- |
| **Image ID** | **Gene Name** | **Symbol** | **Expression Ratio*** |
| 888512 | Small praline-rich protein 2A | Sprr2a | 3.94 |
| 438481 | “adaptor-related protein complex AP-3, sigma 2 subunit” | Ap3s2 | 3.37 |
| 348245 | Keratinocyte differentiation associated protein | Krtdap | 3.34 |
| 581020 | X transporter protein 2 | Xtrp2 | 1.86 |
| 440103 | ectonucleotide pyrophosphatase/phosphodiesterase 2 | Enpp2 | 1.79 |
| 672405 | small proline-rich protein 1A | Sprr1a | 1.7 |
| 1247224 | cathepsin E | Ctse | 1.55 |
| 737373 | "mannose binding lectin, liver (A)" | Mbl1 | 1.53 |
| 776543 | "solute carrier family 2 (facilitated glucose transporter), member 2" | Slc2a2 | 1.51 |
| 570673 | cytoskeletal crystallin | Crym | 1.5 |
| 820137 | CD6 antigen | Cd6 | 1.5 |
| 550993 | "cytochrome P450, 4a10" | Cyp4a10 | 1.42 |
| 762549 | prostate cancer overexpressed gene 1 | Pov1 | 1.41 |
| 465060 | Tripartite motif protein 10 |  | 1.38 |
| 634235 | "casein kinase 1, epsilon" | Csnk1e | 1.34 |
| 472942 | Similar to CREB/ATF family transcription factor, | LOC208677 | 1.31 |
| 680146 | formiminotransferase cyclodeaminase | Ftcd | 1.3 |
| 493244 | "cytochrome P450, 4a14" | Cyp4a14 | 1.27 |
| 888602 | claudin 2 | Cldn2 | 1.21 |
| 656701 | dimethylarginine dimethylaminohydrolase 1 |  | 1.06 |
| 348115 | chloride intracellular channel 3 | Clic3 | 1.02 |
| 335736 | "keratin complex 2, basic, gene 6a" | Krt2-6a | 0.9 |
| 831635 | cholecystokinin | Cck | 0.68 |
| 948648 | "protein phosphatase 1, regulatory (inhibitor) subunit 7" | Ppp1r7 | 0.65 |
| 484289 | "protease, serine, 20" | Prss20-pending, TLSP | 0.61 |
| 1179683 | Similar to chloride channel calcium activated 1 | Clca1 | 0.6 |
| 875031 | casein kappa | LOC232158 | 0.59 |

* The average expression value of tumors carrying Ha-Ras mutation divided by that of tumors with Ha-Ras wild-type in MMTV-Wnt-1 TG mice. p=<0.001. ESTs and riken cDNAs were excluded.
