# Supplementary material for: Changes in gene expression during the development of mammary tumors in MMTV-Wnt-1 transgenic mice
Source: Genome Biol. 2005 Sep 30;6(10):R84. doi: 10.1186/gb-2005-6-10-r84 (PMC1257467; doi:10.1186/gb-2005-6-10-r84)
Supplement: Additional File 3 — A table listing genes differentially expressed between MMTV-Wnt-1-induced tumors in p53-null and in p53-wild-type background [file gb-2005-6-10-r84-S3.doc]

| **Additional data file 3. List of genes differentially expressed between MMTV-Wnt-1-induced tumors in p53-null and in p53-wild-type background** | | | |
| --- | --- | --- | --- |
| **Image ID** | **Gene Name** | **Symbol** | **Expression Ratio*** |
| 572880 | POU domain, class 2, associating factor 1 | Pou2af1 | 3.8 |
| 424433 | cyclin D2 | Ccnd2 | 3.7 |
| 480428 | tweety homolog 1 (Drosophila) | Ttyh1 | 3.7 |
| 721803 | myeloblastosis oncogene | Myb | 2.9 |
| 575702 | myeloblastosis oncogene | Myb | 2.8 |
| 920268 | high mobility group AT-hook 1 | Hmga1 | 2.2 |
| 575698 | 2'-5' oligoadenylate synthetase-like | Oasl2 | 2.2 |
| 464995 | profilin 2 | Pfn2 | 2.2 |
| 694987 | serine (or cysteine) proteinase inhibitor, clade E (nexin, plasminogen activator inhibitor type 1), member 2 | Serpine2 | 2.1 |
| 464497 | serine (or cysteine) proteinase inhibitor, clade E (nexin, plasminogen activator inhibitor type 1), member 2 | Serpine2 | 2 |
| 481151 | procollagen, type IX, alpha 1 | Col9a1 | 2 |
| 634779 | lymphocyte antigen 6 complex, locus D | Ly6d | 2 |
| 733800 | collapsin response mediator protein 5 | Crmp5-pending | 1.9 |
| 425881 | pre B-cell leukemia transcription factor 3 | Pbx3 | 1.8 |
| 444842 | homeo box, msh-like 2 | Msx2 | 1.7 |
| 536464 | phospholipase A2 group VII (platelet-activating factor acetylhydrolase, plasma) | Pla2g7 | 1.7 |
| 747104 | glypican 3 | Gpc3 | 1.7 |
| 440755 | stratifin | Sfn | 1.7 |
| 723118 | B-cell CLL/lymphoma 11A (zinc finger proten) | Bcl11a | 1.5 |
| 425021 | B-cell CLL/lymphoma 11A (zinc finger proten) | Bcl11a | 1.5 |
| 317268 | protease, serine, 20 | Prss20-pending | 0.8 |
| 425427 | potassium intermediate/small conductance calcium-activated channel, subfamily N, member 4 | Kcnn4 | 0.8 |
| 680146 | formiminotransferase cyclodeaminase | Ftcd | 0.8 |
| 777467 | synaptonemal complex protein 3 | Sycp3 | 0.8 |
| 332687 | hypothetical protein MGC7623 | BC002262 | 0.8 |
| 651622 | met proto-oncogene | met | 0.7 |
| 872869 | S100 calcium binding protein A1 | S100a1 | 0.7 |
| 777103 | interleukin 15 receptor, alpha chain |  | 0.7 |
| 425297 | transmembrane protease, serine 2 |  | 0.7 |
| 535409 | alkaline phosphatase 2, liver | Akp2 | 0.7 |
| 351947 | leucine rich repeat protein 1, neuronal | Lrrn1 | 0.6 |
| 639168 | receptor tyr phos.R-C Mm.70 Protein tyrosine phosphatase receptor type C | Rps20 | 0.6 |
| 874383 | lactotransferrin | Ltf | 0.6 |
| 676327 | inter-alpha trypsin inhibitor, heavy chain 2 |  | 0.6 |
| 693315 | CD1d1 antigen | Cd1d1 | 0.6 |
| 465986 | DEAD/H (Asp-Glu-Ala-Asp/His) box polypeptide 26 | Ddx26 | 0.6 |
| 763202 | hypothetical protein | AI385631 | 0.6 |
| 671940 | DNA segment, Chr 16, ERATO Doi 61, expressed |  | 0.5 |
| 695667 | cytochrome P450, 2f2 | Cyp2f2 | 0.5 |
| 904900 | myelin and lymphocyte protein; T-cell differentiation protein | Mal | 0.5 |
| 314215 | Tyrosine aminotransferase | Tat | 0.5 |
| 721831 | CD59a antigen | Cd59a | 0.5 |
| 635299 | Mus musculus hemogen-1 mRNA, complete cds | Hemgn | 0.5 |
| 765935 | cholinergic receptor, nicotinic, beta polypeptide 1 (muscle) | Chrnb1 | 0.5 |
| 777655 | popeye 2 | Pop2-pending | 0.5 |
| 775858 | prolactin receptor related sequence 1 |  | 0.5 |
| 355112 | ral guanine nucleotide dissociation stimulator | Rgds | 0.5 |
| 373793 | calponin 1 | Cnn1 | 0.4 |
| 620870 | lysosomal acid lipase 1 | 2610301D06Rik | 0.4 |
| 595848 | immunoglobulin joining chain | Igj | 0.4 |
| 751756 | CD14 antigen | Cd14 | 0.4 |
| 618910 | lysosomal acid lipase 1 | 2610301D06Rik | 0.4 |
| 832714 | retinoblastoma 1 | Rb1 | 0.4 |
| 596470 | CD79B antigen | Cd79b | 0.4 |
| 850206 | phospholipid scramblase 2 | Plscr2 | 0.4 |
| 480893 | peroxisome biogenesis factor 16 | Pex16 | 0.4 |
| 418633 | macrophage galactose N-acetyl-galactosamine specific lectin | Mgl1 | 0.4 |
| 464679 | alpha-2-HS-glycoprotein |  | 0.3 |
| 459676 | zinc finger protein, subfamily 1A, 1 (Ikaros) |  | 0.3 |
| 571759 | secreted phosphoprotein 1 | Spp1 | 0.1 |

*The average expression value of tumors in MMTV-Wnt-1/p53-/- mice divided by that of tumors in MMTV-Wnt-1/p53+/+ mice. p=<0.001. ESTs and riken cDNAs were excluded.
