# Supplementary material for: Changes in gene expression during the development of mammary tumors in MMTV-Wnt-1 transgenic mice
Source: Genome Biol. 2005 Sep 30;6(10):R84. doi: 10.1186/gb-2005-6-10-r84 (PMC1257467; doi:10.1186/gb-2005-6-10-r84)
Supplement: Additional File 4 — A table listing genes differentially expressed between tumors from MMTV-Wnt-1 transgenic/Pten+/- mice with loss of heterozygosity at the Pten locus and tumors from Wnt-1 transgenic/Pten+/+ mice [file gb-2005-6-10-r84-S4.doc]

| **Additional data file 4. List of genes differentially expressed between tumors from MMTV-Wnt-1 TG/Pten+/- mice with LOH at Pten locus and tumors from Wnt-1 TG/Pten+/+ mice** | | | |
| --- | --- | --- | --- |
| **Image ID** | **Gene name** | **Symbol** | **Expression Ratio*** |
| 476703 | meiotic cohesion Rec8 | mrec8 | 4.91 |
| 493282 | placental growth factor | Pgf | 3.94 |
| 571367 | apoptosis NIP3 | Bnip3 | 3.53 |
| 752301 | 2'-5' oligoadenylate synthetase 1A | Oas1a | 3.02 |
| 463388 | BCL2/adenovirus E1B 19 kDa-interacting protein 1, NIP3 | Bnip3 | 2.93 |
| 575698 | 2'-5' oligoadenylate synthetase-like | Oasl2 | 2.83 |
| 779373 | hypoxia induced gene 1 | Hig1-pending | 2.64 |
| 599128 | myxovirus (influenza virus) resistance 2 | Mx2 | 2.51 |
| 737364 | polymeric immunoglobulin receptor | Pigr | 2.23 |
| 641058 | nuclear factor of kappa light chain gene enhancer in B-cells inhibitor, alpha | Nfkbia | 2.21 |
| 776890 | argininosuccinate synthetase 1 | Ass1 | 2.16 |
| 903419 | aldolase 1, A isoform | Aldo1 | 2.02 |
| 353366 | synuclein, alpha | Snca | 1.92 |
| 418952 | insulin-like growth factor binding protein 5 | Igfbp5 | 1.79 |
| 738252 | CCAAT/enhancer binding protein (C/EBP), alpha | Cebpa | 1.76 |
| 656654 | aquaporin 1 | Aqp1 | 1.66 |
| 422205 | interferon-stimulated protein (20 kDa) | Isg20 | 1.6 |
| 439199 | aminolevulinic acid synthase 2, erythroid |  | 1.56 |
| 599164 | thymidylate kinase family LPS-inducible member | Tyki | 1.49 |
| 329940 | carbonic anhydrase 6 |  | 1.47 |
| 680815 | lectin, galactose binding, soluble 9 | Lgals9 | 1.44 |
| 318134 | translocator of inner mitochondrial membrane 17 kDa, a | Timm17a | 1.33 |
| 669813 | S100 calcium binding protein A8 (calgranulin A) | S100a8 | 1.25 |
| 718248 | Mus musculus Kell protein (Kel) mRNA, complete cds | Kel | 0.89 |
| 467172 | parathyroid hormone receptor | Pthr | 0.81 |
| 639168 | receptor tyr phos.R-C Mm.70 Protein tyrosine phosphatase receptor type C | Rps20 | 0.79 |
| 533003 | stromal cell derived factor 1 |  | 0.69 |
| 719034 | phospholipase C-like protein | Plce1 | 0.62 |
| 873849 | cubilin (intrinsic factor-cobalamin receptor) | Cubn | 0.6 |
| 678748 | hydroxyacid oxidase 1, liver | Hao1 | 0.59 |
| 477066 | four and a half LIM domains 1 | Fhl1 | 0.58 |
| 851201 | complement component 2 (within H-2S) | C2 | 0.56 |
| 622274 | membrane-spanning 4-domains, subfamily A, member 2 | Ms4a1 | 0.56 |
| 439383 | slit homolog 3 (Drosophila) |  | 0.55 |
| 313322 | insulin-like growth factor 1 | Igf1 | 0.55 |
| 636695 | immunoglobulin heavy chain 1 (serum IgG2a) | Igh-1 | 0.55 |
| 777655 | popeye 2 | Pop2-pending | 0.54 |
| 386417 | tensin | Tns | 0.53 |
| 480620 | procollagen, type VI, alpha 3 | Col6a3 | 0.52 |
| 445565 | endothelial-specific receptor tyrosine kinase |  | 0.49 |
| 920211 | solute carrier family 39 (iron-regulated transporter), member 1 | Slc39a1 | 0.48 |
| 676176 | complement component 1, s subcomponent | C1s | 0.48 |
| 352450 | procollagen, type VI, alpha 1 | Col6a1 | 0.48 |
| 403499 | Fc receptor, IgG, low affinity III | Fcgr3 | 0.47 |
| 551003 | IG alpha chain C region |  | 0.42 |
| 735607 | cDNA sequence AF155546 | AF155546 | 0.42 |
| 617816 | complement component 1, r subcomponent |  | 0.41 |
| 367435 | biglycan | Bgn | 0.39 |
| 719592 | GATA-binding protein 2 | Gata2 | 0.38 |
| 638662 | biglycan | Bgn | 0.34 |
| 777018 | selenoprotein P, plasma, 1 | Sepp1 | 0.32 |
| 331457 | Legumain | Lgmn | 0.32 |
| 749361 | chemokine (C-C) receptor 2 | Ccr2 | 0.32 |
| 333232 | blocked early in transport 1 homolog (S. Cerevisiae) | Bet1 | 0.29 |
| 747378 | butyrophilin-like 2 |  | 0.24 |
| 749660 | IG alpha chain C region |  | 0.24 |

*The average expression value of tumors from MMTV-Wnt-1 TG/Pten+/- mice with LOH divided by that of tumors from MMTV-Wnt-1 TG/Pten+/+ mice. p=<0.001. ESTs and riken cDNAs were excluded.
