# Supplementary material for: Changes in gene expression during the development of mammary tumors in MMTV-Wnt-1 transgenic mice
Source: Genome Biol. 2005 Sep 30;6(10):R84. doi: 10.1186/gb-2005-6-10-r84 (PMC1257467; doi:10.1186/gb-2005-6-10-r84)
Supplement: Additional File 5 — A table listing genes differentially expressed between virgin mammary glands from nontransgenic mice and hyperplastic mammary glands from MMTV-Wnt-1 transgenic mice [file gb-2005-6-10-r84-S5.doc]

| **Additional data file 5. List of genes that are differentially expressed between virgin mammary glands from non-TG mice and hyperplastic mammary glands from MMTV-Wnt-1 TG mice** | | | |
| --- | --- | --- | --- |
| **Image ID** | **Gene Name** | **Symbol** | **Expression Ratio*** |
| 717457 | fibroblast growth factor binding protein 1 | Fgfbp1 | 12.34 |
| 719965 | desmocollin 2 | Dsc2 | 7.5 |
| 355929 | inhibitor of DNA binding 4 | Idb4 | 6.69 |
| 403869 | crystallin, beta A4 | Cryba4 | 6.19 |
| 442048 | stimulated by retinoic acid gene 6 | Stra6 | 5.9 |
| 776007 | major urinary protein 2 | Mup2 | 5.79 |
| 672405 | small proline-rich protein 1A | Sprr1a | 5.43 |
| 699460 | Indian hedgehog | Ihh | 5.07 |
| 837565 | Kruppel-like factor 5 | Klf5 | 4.77 |
| 404510 | expressed sequence AA987140 | Stra6 | 4.65 |
| 717226 | lectin, galactose binding, soluble 3 | Lgals3 | 3.83 |
| 716630 | stathmin 1 | Lag | 3.81 |
| 1195103 | FXYD domain-containing ion transport regulator 3 | Fxyd3 | 3.7 |
| 676349 | butyrobetaine (gamma), 2-oxoglutarate dioxygenase 1 (gamma-butyrobetaine hydroxylase) |  | 3.68 |
| 776133 | cadherin 1 | Cdh1 | 3.64 |
| 443870 | keratin complex 2, basic, gene 7 |  | 3.62 |
| 441346 | myelocytomatosis oncogene | Myc | 3.56 |
| 481198 | tumor-associated calcium signal transducer 2 | Tacstd2 | 3.46 |
| 747136 | high mobility group box 2 | Hmgb2 | 3.36 |
| 335736 | keratin complex 2, basic, gene 6a | Krt2-6a | 3.35 |
| 481011 | tripartite motif protein 29 | Trim29 | 3.35 |
| 484261 | keratin complex 1, acidic, gene 13 | Krt1-13 | 2.99 |
| 372468 | ladinin |  | 2.98 |
| 406897 | retinol binding protein 1, cellular | Rbp1 | 2.93 |
| 805046 | DNA segment, Chr 7, ERATO Doi 684, expressed | D7Ertd684e | 2.85 |
| 419886 | transcription factor AP-2, gamma | Tcfap2c | 2.77 |
| 493658 | lipocalin 2 | Lcn2 | 2.77 |
| 441290 | tripartite motif protein 29 | Trim29 | 2.73 |
| 598827 | aryl-hydrocarbon receptor | Ahr | 2.73 |
| 1447403 | WASP family 1 | Wasf1 | 2.71 |
| 463860 | platelet-derived growth factor, C polypeptide | Pdgfc | 2.67 |
| 697383 | RAB25, member RAS oncogene family | Rab25 | 2.63 |
| 420975 | erythrocyte protein band 4.1-like 4b | Epb4.1l4b | 2.6 |
| 474107 | tumor necrosis factor receptor superfamily, member 19 | Tnfrsf19 | 2.56 |
| 762437 | membrane-associated protein 17 | Map17-pending | 2.55 |
| 656701 | dimethylarginine dimethylaminohydrolase 1 | Ddah1 | 2.52 |
| 427314 | cartilage derived retinoic acid sensitive protein | Cdrap | 2.47 |
| 482641 | RNA, U22 small nucleolar | Rnu22 | 2.46 |
| 1348179 | ribosomal protein L12 | Rpl12 | 2.41 |
| 920235 | RAB25, member RAS oncogene family | Rab25 | 2.28 |
| 1383428 | expressed sequence AI462521 | G6pdx | 2.28 |
| 466295 | ribosomal protein S15 | Rps15 | 2.27 |
| 891131 | eosinophil-associated ribonuclease 2 | Ear2 | 2.26 |
| 336325 | connective tissue growth factor | Ctgf | 2.19 |
| 524442 | ribosomal protein L7 | Rpl7 | 2.19 |
| 1178990 | expressed sequence AI645535 | Map2k7 | 2.18 |
| 442662 | eukaryotic translation elongation factor 1 beta 2 | Eef1b2 | 2.17 |
| 318642 | popeye 3 | Pop3-pending | 2.16 |
| 525168 | Mpv17 transgene, kidney disease mutant-like | Mpv17l | 2.16 |
| 317268 | protease, serine, 20 | Prss20-pending | 2.14 |
| 572463 | potassium channel, subfamily K, member 5 | Kcnk5 | 2.14 |
| 1178689 | chitinase 3-like 1 | Chi3l1 | 2.12 |
| 949520 | frizzled homolog 7 (Drosophila) | Fzd7 | 2.11 |
| 570673 | crystallin, mu | Crym | 2.05 |
| 678793 | pituitary tumor-transforming 1 | Pttg1 | 2.04 |
| 400741 | laminin receptor 1 (67kD, ribosomal protein SA) | Lamr1 | 1.92 |
| 335883 | paraoxonase 1 | Pon1 | 1.89 |
| 423942 | wingless-related MMTV integration site 5B | Wnt5b | 1.83 |
| 1398634 | Small inducible cytokine subfamily A17 | Scya17 | 0.69 |
| 1399486 | sorting nexin 17 | D5Ertd260e | 0.58 |
| 695845 | integrin alpha 7 | Itga7 | 0.55 |
| 766704 | glycerol kinase | Gyk | 0.55 |
| 935463 | glycerol kinase | Gyk | 0.54 |
| 832113 | adipose differentiation related protein | Adfp | 0.54 |
| 439199 | aminolevulinic acid synthase 2, erythroid | Alas2 | 0.53 |
| 1245198 | Notch gene homolog 1, (Drosophila) | Notch1 | 0.53 |
| 390390 | kininogen | Kng | 0.52 |
| 483333 | acetyl-Coenzyme A dehydrogenase, medium chain | Acadm | 0.52 |
| 831964 | tissue inhibitor of metalloproteinase 2 | Timp2 | 0.52 |
| 776048 | ceroid-lipofuscinosis, neuronal 2 | Cln2 | 0.52 |
| 875183 | membrane-associated tyrosine-and threonine-specific cdc2-inhibitory kinase | Pkmyt1-pending | 0.52 |
| 1313962 | tissue inhibitor of metalloproteinase 4 |  | 0.52 |
| 336159 | histocompatibility 2, complement component factor B | H2-Bf | 0.51 |
| 575665 | protein tyrosine phosphatase, non-receptor type 16 | Ptpn16 | 0.51 |
| 638394 | frequently rearranged in advanced T-cell lymphomas | Frat1 | 0.51 |
| 806940 | A kinase (PRKA) anchor protein (gravin) 12 | Akap12 | 0.51 |
| 820132 | LIM only 2 | Lmo2 | 0.51 |
| 891344 | ribosomal protein L27a | Rpl27a | 0.51 |
| 1178993 | SH3-domain GRB2-like B1 (endophilin) | Sh3glb1 | 0.51 |
| 1245154 | interleukin 3 receptor, alpha chain | Il3ra | 0.51 |
| 891366 | Socs-5 | Socs5 | 0.51 |
| 483650 | Kruppel-like factor 15 | Klf15 | 0.5 |
| 677093 | C-terminal binding protein 1 | Ctbp1 | 0.5 |
| 734810 | transient receptor potential cation channel, subfamily C, member 4 associated protein | Trrp4ap-pending | 0.5 |
| 761668 | ferritin heavy chain | Fth | 0.5 |
| 803416 | N-myc downstream regulated 1 | Ndr1 | 0.5 |
| 776250 | renin 2 tandem duplication of Ren1 | Ren1 | 0.5 |
| 1054503 | nuclear receptor subfamily 1, group I, member 3 | Nr1i3 | 0.5 |
| 804313 | EGL nine homolog 1 (C. elegans) | Egln1 | 0.49 |
| 437290 | membrane protein, palmitoylated (55 kDa) | Mpp1 | 0.49 |
| 735186 | nuclear receptor binding factor 1 | Nrbf1 | 0.49 |
| 766482 | retinal short-chain dehydrogenase/reductase 1 | Rsdr1-pending | 0.49 |
| 533003 | stromal cell derived factor 1 | Sdf1 | 0.48 |
| 533314 | staufen (RNA binding protein) homolog 2 (Drosophila) | Stau2 | 0.48 |
| 523945 | developmentally regulated GTP binding protein 2 | Drg2 | 0.48 |
| 850720 | transmembrane protease, serine 2+D147 | Tmprss2 | 0.48 |
| 891323 | cDNA sequence BC004044 | MGC7673 | 0.48 |
| 1245498 | Tnfa-induced adipose-related protein | Tiarp-pending | 0.48 |
| 1314017 | isocitrate dehydrogenase 3 (NAD+) alpha | 1500012E04Rik | 0.48 |
| 851311 | nuclear receptor binding protein |  | 0.47 |
| 735225 | eukaryotic translation elongation factor 1 alpha 1 | Eef1a1 | 0.47 |
| 535388 | engulfment and cell motility 2, ced-12 homolog (C. elegans) | 1190002F24Rik | 0.47 |
| 405994 | X-prolyl aminopeptidase (aminopeptidase P) 1, soluble |  | 0.47 |
| 550702 | hypothetical protein MGC38336 | Birc4 | 0.47 |
| 1245142 | procollagen, type IV, alpha 6 | Col4a6 | 0.47 |
| 1347718 | calcium binding protein, 140 kDa | Cab140 | 0.47 |
| 920215 | mitochondrial carrier homolog 2 | Mtch2-pending | 0.46 |
| 864344 | monocyte to macrophage differentiation-associated | Mmd | 0.46 |
| 717095 | malate dehydrogenase, mitochondrial | Mor1 | 0.46 |
| 766441 | sideroflexin 1 | Sfxn1 | 0.46 |
| 521951 | Williams-Beuren syndrome chromosome region 14 homolog (human) | Wbscr14 | 0.46 |
| 851802 | heat shock 27kD protein 3 | Hspb3 | 0.46 |
| 1230469 | epsin 2 | Epn2 | 0.46 |
| 1068786 | DEAD/H (Asp-Glu-Ala-Asp/His) box polypeptide 50 | GU2 | 0.46 |
| 404615 | benzodiazepine receptor, peripheral | Bzrp | 0.45 |
| 426965 | crystallin, alpha C | Cryac | 0.45 |
| 439814 | early B-cell factor 1 | Ebf | 0.45 |
| 440103 | ectonucleotide pyrophosphatase/phosphodiesterase 2 | Enpp2 | 0.45 |
| 922965 | ring finger protein 11 | Rnf11 | 0.45 |
| 776210 | glucose phosphate isomerase 1 | Gpi1 | 0.45 |
| 1179017 | frizzled homolog 4 (Drosophila) | Fzd4 | 0.45 |
| 1196280 | acetyl-Coenzyme A dehydrogenase, long-chain | Acadl | 0.45 |
| 963099 | isovaleryl coenzyme A dehydrogenase | Ivd | 0.45 |
| 469047 | ATP synthase mitochondrial F1 complex assembly factor 2 |  | 0.44 |
| 478848 | discoidin domain receptor family, member 2 | Ddr2 | 0.44 |
| 574792 | microsomal glutathione S-transferase 3 | 2010306B17Rik | 0.44 |
| 579715 | glyceraldehyde-3-phosphate dehydrogenase | Gapd | 0.44 |
| 697010 | transcobalamin 2 | Tcn2 | 0.44 |
| 948547 | hypothetical protein LOC224105 | D16Ertd269e | 0.44 |
| 1179233 | carbohydrate (keratan sulfate Gal-6) sulfotransferase 1 | Chst1 | 0.44 |
| 1247470 | Tnfa-induced adipose-related protein | Tiarp-pending | 0.44 |
| 949230 | N-myc downstream regulated 2 | Ndr2 | 0.44 |
| 353366 | synuclein, alpha | Snca | 0.43 |
| 482847 | uncoupling protein 3, mitochondrial | Ucp3 | 0.43 |
| 889543 | heat shock protein, 74 kDa, A | Hspa9a | 0.43 |
| 671205 | aminolevulinate, delta-, dehydratase | Alad | 0.43 |
| 734688 | melanoma cell adhesion molecule | Mcam | 0.43 |
| 1511643 | CD36 antigen | Cd36 | 0.43 |
| 1495273 | surfactant associated protein D | Sftpd | 0.43 |
| 403656 | sorbin and SH3 domain containing 1 | Sh3d5 | 0.42 |
| 480467 | aquaporin 1 | Aqp1 | 0.42 |
| 481934 | electron transferring flavoprotein, alpha polypeptide | D9Ertd394e | 0.42 |
| 948648 | protein phosphatase 1, regulatory (inhibitor) subunit 7 | Ppp1r7 | 0.42 |
| 672972 | glutathione transferase zeta 1 (maleylacetoacetate isomerase) | Gstz1 | 0.42 |
| 736299 | staufen (RNA binding protein) homolog 1 (Drosophila) | Stau1 | 0.42 |
| 776036 | branched chain aminotransferase 2, mitochondrial | Bcat2 | 0.42 |
| 732889 | inner mitochondrial membrane peptidase 2-like (S. cerevisiae) | Immp2l-pending | 0.42 |
| 832109 | resistin | Retn | 0.42 |
| 851134 | adiponutrin | Adpn-pending | 0.42 |
| 851374 | stearoyl-Coenzyme A desaturase 1 | Scd1 | 0.42 |
| 1248540 | solute carrier family 1, member 7 | Slc1a7 | 0.42 |
| 1364620 | solute carrier family 25 (mitochondrial deoxynucleotide carrier), member 19 | 2900089E13Rik | 0.42 |
| 876922 | ATP-binding cassette, sub-family D (ALD), member 4 | Abcd4 | 0.42 |
| 1383598 | heterogeneous nuclear ribonucleoprotein H1 | Hnrph1 | 0.42 |
| 334182 | amyotrophic lateral sclerosis 2 (juvenile) homolog (human) | 3222402C23Rik | 0.41 |
| 332285 | carbonic anhydrase 4 | Car4 | 0.41 |
| 353456 | early B-cell factor 1 | Ebf | 0.41 |
| 477905 | DNA segment, Chr 14, University of California at Los Angeles 2 | D14Ucla2 | 0.41 |
| 579391 | carbonic anhydrase 2 | Car2 | 0.41 |
| 581125 | G protein gamma 3 linked gene | Gng3lg | 0.41 |
| 596447 | histocompatibility 2, Q region locus 7 | H2-Q7 | 0.41 |
| 634846 | sphingosine kinase 1 | Sphk1 | 0.41 |
| 721051 | peroxisome proliferator activated receptor gamma | Pparg | 0.41 |
| 1314756 | calpain 1 | Capn1 | 0.41 |
| 335369 | arachidonate 12-lipoxygenase, 12R type | Alox12b | 0.4 |
| 402641 | alpha thalassemia/mental retardation syndrome X-linked homolog (human) | Xnp | 0.4 |
| 475944 | ectonucleotide pyrophosphatase/phosphodiesterase 5 | Enpp5 | 0.4 |
| 478168 | pleiotrophin | Ptn | 0.4 |
| 484299 | endoglin | Eng | 0.4 |
| 619563 | solute carrier family 1, member 7 | Slc1a7 | 0.4 |
| 776543 | solute carrier family 2 (facilitated glucose transporter), member 2 | Slc2a2 | 0.4 |
| 766446 | regulator of G-protein signaling 3 | Rgs3 | 0.4 |
| 890764 | CD1d1 antigen | Cd1d1 | 0.4 |
| 1247536 | branched chain ketoacid dehydrogenase E1, alpha polypeptide | Bckdha | 0.4 |
| 1265861 | adiponutrin | Adpn-pending | 0.4 |
| 350180 | thioredoxin interacting protein | Vdup1-pending | 0.39 |
| 478782 | protein kinase, cAMP dependent, catalytic, alpha | Prkaca | 0.39 |
| 480893 | peroxisome biogenesis factor 16 | Pex16 | 0.39 |
| 523123 | ectonucleotide pyrophosphatase/phosphodiesterase 5 | Enpp5 | 0.39 |
| 832242 | plasmalemma vesicle associated protein | Plvap | 0.39 |
| 875530 | monoglyceride lipase | Mgll | 0.39 |
| 1245766 | parvalbumin | Pva | 0.39 |
| 775189 | isocitrate dehydrogenase 3 (NAD+) alpha | 1500012E04Rik | 0.38 |
| 317156 | propionyl Coenzyme A carboxylase, beta polypeptide | Pccb | 0.38 |
| 350336 | peroxisome biogenesis factor 16 | Pex16 | 0.38 |
| 463388 | BCL2/adenovirus E1B 19 kDa-interacting protein 1, NIP3 | Bnip3 | 0.38 |
| 676636 | carboxypeptidase B2 (plasma) | Cpb2 | 0.38 |
| 734064 | RAB5C, member RAS oncogene family | Rab5c | 0.38 |
| 777018 | selenoprotein P, plasma, 1 | Sepp1 | 0.38 |
| 419756 | erythrocyte protein band 7.2 | Epb7.2 | 0.37 |
| 442843 | glutamate dehydrogenase | Glud | 0.37 |
| 571367 | BCL2/adenovirus E1B 19 kDa-interacting protein 1, NIP3 | Bnip3 | 0.37 |
| 581101 | phenylalanine hydroxylase | Pah | 0.37 |
| 831959 | phospholipase A2, activating protein | Plaa | 0.37 |
| 737492 | secretory carrier membrane protein 3 | Scamp3 | 0.37 |
| 1247588 | adipocyte complement related protein of 30 kDa | Acrp30 | 0.37 |
| 1314022 | lipoprotein lipase | Lpl | 0.37 |
| 1244469 | regulator of G-protein signaling 5 | Rgs5 | 0.37 |
| 338005 | apolipoprotein A-V | Apoa5 | 0.36 |
| 466208 | retinoid X receptor gamma | Rxrg | 0.36 |
| 891203 | leptin | Lep | 0.36 |
| 693315 | CD1d1 antigen | Cd1d1 | 0.36 |
| 699181 | solute carrier family 25 (mitochondrial carnitine/acylcarnitine translocase), member 20 | Slc25a20 | 0.36 |
| 832584 | Fc receptor, IgG, low affinity III | Fcgr3 | 0.36 |
| 336021 | inner membrane protein, mitochondrial | 1700082C19Rik | 0.35 |
| 332442 | brain protein 44-like | Brp44l | 0.35 |
| 831665 | fat specific gene 27 | Fsp27 | 0.35 |
| 820177 | amine oxidase, copper containing 3 | Aoc3 | 0.35 |
| 1195467 | fatty acid synthase | Fasn | 0.35 |
| 1177633 | high mobility group 20A | Hmg20a | 0.35 |
| 313426 | catenin beta | Catnb | 0.34 |
| 318134 | translocator of inner mitochondrial membrane 17 kDa, a | Timm17a | 0.34 |
| 386417 | tensin | Tns | 0.34 |
| 920327 | carbohydrate sulfotransferase 2 | Chst2 | 0.34 |
| 721831 | CD59a antigen | Cd59a | 0.34 |
| 388288 | solute carrier family 25 (mitochondrial carrier; dicarboxylate transporter), member 10 | Slc25a10 | 0.33 |
| 436894 | fatty acid binding protein 7, brain | Fabp7 | 0.33 |
| 572379 | solute carrier family 27 (fatty acid transporter), member 2 | Slc27a2 | 0.33 |
| 888842 | pyruvate dehydrogenase E1 alpha 1 | Pdha1 | 0.33 |
| 338088 | enoyl coenzyme A hydratase 1, peroxisomal | Ech1 | 0.32 |
| 330336 | histocompatibility 2, K region | H2-K | 0.32 |
| 903419 | aldolase 1, A isoform | Aldo1 | 0.32 |
| 331186 | caveolin, caveolae protein, 22 kDa | Cav | 0.31 |
| 483688 | 3-hydroxy-3-methylglutaryl-Coenzyme A synthase 1 | Pbx1 | 0.31 |
| 890429 | carboxylesterase 3 | Ces3 | 0.31 |
| 1448821 | fatty acid binding protein 4, adipocyte | Fabp4 | 0.31 |
| 949505 | carbonic anhydrase 13 | Car13 | 0.31 |
| 473778 | pyruvate carboxylase | Pcx | 0.3 |
| 596968 | caveolin, caveolae protein, 22 kDa | Cav | 0.3 |
| 890915 | peroxisome proliferator activated receptor gamma | Pparg | 0.3 |
| 764542 | epoxide hydrolase 2, cytoplasmic | Ephx2 | 0.3 |
| 656654 | aquaporin 1 | Aqp1 | 0.3 |
| 1314739 | carbonic anhydrase 3 | Car3 | 0.3 |
| 1347411 | positive cofactor 2, multiprotein complex, glutamine/Q-rich-associated protein | Pcqap | 0.3 |
| 419437 | nidogen 1 | Nid1 | 0.29 |
| 479750 | adenylate kinase 3 alpha-like | Akl3l-pending | 0.29 |
| 641512 |  | Ncf4 | 0.29 |
| 935524 | regulator of G-protein signaling 5 | Rgs5 | 0.29 |
| 949423 | acetyl-Coenzyme A synthetase 2 (ADP forming) | Acas1 | 0.29 |
| 831701 | transcription factor 1 | Tcf1 | 0.29 |
| 820409 | pyruvate dehydrogenase E1 alpha 1 | Pdha1 | 0.29 |
| 1245404 | lipoprotein lipase | Lpl | 0.29 |
| 329741 | angiopoietin-like 4 | Angptl4 | 0.28 |
| 444027 | heat shock protein, 74 kDa, A | Hspa9a | 0.28 |
| 747583 | NCK-associated protein 1 | Nckap1 | 0.28 |
| 475796 | isocitrate dehydrogenase 3 (NAD+) alpha | 1500012E04Rik | 0.27 |
| 483777 | diacylglycerol O-acyltransferase 1 | Dgat1 | 0.27 |
| 679163 | regulator of G-protein signaling 5 | Rgs5 | 0.27 |
| 1346958 | regulator of G-protein signaling 5 | Rgs5 | 0.27 |
| 314442 | fumarylacetoacetate hydrolase | Fah | 0.26 |
| 482369 | ATP-binding cassette, sub-family A (ABC1), member 1 | Abca1 | 0.26 |
| 313859 | diacylglycerol O-acyltransferase 1 | Dgat1 | 0.25 |
| 329884 | citrate synthase | Cs | 0.25 |
| 747880 | acyl-Coenzyme A oxidase 1, palmitoyl | Acox1 | 0.25 |
| 678863 | esterase 22 |  | 0.24 |
| 1265649 | malic enzyme, supernatant | Mod1 | 0.22 |
| 864409 | CD36 antigen | Cd36 | 0.21 |
| 1398011 | guanine nucleotide binding protein (G protein), gamma 10 | Gng10 | 0.21 |
| 335220 | monoglyceride lipase | Mgll | 0.19 |
| 776426 | pleiotropic regulator 1, PRL1 homolog (Arabidopsis) | Plrg1 | 0.19 |
| 579349 | epoxide hydrolase 2, cytoplasmic | Ephx2 | 0.18 |
| 570675 | glycerol phosphate dehydrogenase 1, cytoplasmic adult | Gdc1 | 0.17 |
| 576881 | fatty acid synthase | Fasn | 0.14 |
| 820409 | pyruvate dehydrogenase E1 alpha 1 | Pdha1 | 0.14 |
| 351557 | cell death-inducing DNA fragmentation factor, alpha subunit-like effector A | Cidea | 0.1 |

*The average expression value of hyperplastic mammary glands from MMTV-Wnt-1 TG mice divided by that of virgin mammary glands from non-TG mice. p=<0.001. ESTs and riken cDNAs were excluded.
