# Supplementary material for: Changes in gene expression during the development of mammary tumors in MMTV-Wnt-1 transgenic mice
Source: Genome Biol. 2005 Sep 30;6(10):R84. doi: 10.1186/gb-2005-6-10-r84 (PMC1257467; doi:10.1186/gb-2005-6-10-r84)
Supplement: Additional File 6 — A table listing genes expressed three-fold or higher in fat tissues than in mammary tumors from MMMTV-Wnt-1 and MMTV-Neu transgenic mice [file gb-2005-6-10-r84-S6.doc]

| **Additional data file 6. List of genes expressed 3 fold or higher in fat tissues than in mammary tumors from MMMTV-Wnt-1 and MMTV-Neu TG mice.** | | | |
| --- | --- | --- | --- |
| **Image ID** | **Gene name** | **Symbol** | **Expression Ratio*** |
| 317156 | propionyl Coenzyme A carboxylase, beta polypeptide | Pccb | 0.33 |
| 331264 | twist gene homolog, (Drosophila) | Twist | 0.33 |
| 401608 | AXL receptor tyrosine kinase | Axl | 0.33 |
| 573301 | CD97 antigen | Cd97 | 0.33 |
| 620221 | CUG triplet repeat,RNA binding protein 2 | Cugbp2 | 0.33 |
| 637493 | torsin family 2, member A | Tor2a | 0.33 |
| 890607 | endomucin | Emcn-pending | 0.33 |
| 949564 | presenilin 2 | Psen2 | 0.33 |
| 671205 | aminolevulinate, delta-, dehydratase | Alad | 0.33 |
| 851201 | complement component 2 (within H-2S) | C2 | 0.33 |
| 1245498 | Tnfa-induced adipose-related protein | Tiarp-pending | 0.33 |
| 1195716 | tweety homolog 2 (Drosophila) | Ttyh2 | 0.33 |
| 1247073 | fibroblast growth factor receptor-like 1 | Fgfrl1 | 0.33 |
| 408233 | potassium intermediate/small conductance calcium-activated channel, subfamily N, member 1 | Kcnn1 | 0.32 |
| 438581 | intercellular adhesion molecule 2 | Icam2 | 0.32 |
| 851311 | nuclear receptor binding protein | Nrbp | 0.32 |
| 902923 | tissue inhibitor of metalloproteinase 2 | Timp2 | 0.32 |
| 948842 | DNA segment, Chr 17, ERATO Doi 197, expressed | D17Ertd197e | 0.32 |
| 693718 | presenilin 2 | Psen2 | 0.32 |
| 831964 | tissue inhibitor of metalloproteinase 2 |  | 0.32 |
| 820240 | protein-tyrosine sulfotransferase 1 | Tpst1 | 0.32 |
| 1066909 | laminin, alpha 4 | Lama4 | 0.32 |
| 1349715 | ATP-binding cassette, sub-family A (ABC1), member 1 | Abca1 | 0.32 |
| 876922 | "ATP-binding cassette, sub-family D (ALD), member 4" |  | 0.32 |
| 949230 | N-myc downstream regulated 2 | Ndr2 | 0.32 |
| 385220 | epimorphin | Epim | 0.31 |
| 472801 | microfibrillar associated protein 5 | Mfap5-pending | 0.31 |
| 476416 | CD151 antigen | Cd151 | 0.31 |
| 480620 | procollagen, type VI, alpha 3 | Col6a3 | 0.31 |
| 598956 | RIKEN cDNA 0610037F22 gene | 0610037F22Rik | 0.31 |
| 583638 | aminolevulinic acid synthase 1 | Alas1 | 0.31 |
| 679896 | dipeptidylpeptidase 4 | Dpp4 | 0.31 |
| 1364620 | solute carrier family 25 (mitochondrial deoxynucleotide carrier), member 19 | Slc25a19 | 0.31 |
| 948474 | RIKEN cDNA 2610205H19 gene | 2610205H19Rik | 0.31 |
| 402641 | alpha thalassemia/mental retardation syndrome X-linked homolog (human) | Atrx | 0.3 |
| 639481 | insulin-like growth factor binding protein 3 | Igfbp3 | 0.3 |
| 640099 | tyrosine kinase, non-receptor, 2 | Tnk2 | 0.3 |
| 850127 | synaptonemal complex protein 3 | Sycp3 | 0.3 |
| 876351 | adiponutrin | Adpn | 0.3 |
| 876313 | similar to Mid-1-related chloride channel 1 | LOC229725 | 0.3 |
| 891131 | eosinophil-associated ribonuclease 2 | Ear2 | 0.3 |
| 1066878 | vacuolar protein sorting 16 (yeast) | Vps16 | 0.3 |
| 1382855 | amyloid beta (A4) precursor-like protein 2 | Aplp2 | 0.3 |
| 1510870 | lysosomal trafficking regulator | Lyst | 0.3 |
| 876149 | RIKEN cDNA E330036I19 gene | E330036I19Rik | 0.3 |
| 949505 | carbonic anhydrase 13 | Car13 | 0.3 |
| 439639 | RAB3D, member RAS oncogene family | Rab3d | 0.29 |
| 477541 | acupuncture induced gene 1 | Aig1-pending | 0.29 |
| 935557 | protein tyrosine phosphatase, non-receptor type 9 | Ptpn9 | 0.29 |
| 523123 | ectonucleotide pyrophosphatase/phosphodiesterase 5 | Enpp5 | 0.29 |
| 949821 | transmembrane 4 superfamily member 2 | Tm4sf2 | 0.29 |
| 891061 | erythrocyte protein band 4.1-like 1 | Epb4.1l1 | 0.29 |
| 1398066 | cDNA sequence BC002292 | BC002292 | 0.29 |
| 426546 | annexin A5 | Anxa5 | 0.28 |
| 437290 | membrane protein, palmitoylated (55 kDa) | Mpp1 | 0.28 |
| 442243 | "ral guanine nucleotide dissociation stimulator,-like 1" |  | 0.28 |
| 479895 | platelet derived growth factor receptor, alpha polypeptide | Pdgfra | 0.28 |
| 679163 | regulator of G-protein signaling 5 | Rgs5 | 0.28 |
| 720207 | leucine-rich alpha-2-glycoprotein | Lrg-pending | 0.28 |
| 849845 | guanine nucleotide binding protein, alpha inhibiting 1 | Gnai1 | 0.28 |
| 862970 | inositol polyphosphate-5-phosphatase, 72 kDa | Inpp5e | 0.28 |
| 874497 | SRY-box containing gene 10 | Sox10 | 0.28 |
| 1054017 | peroxisome proliferator activated receptor gamma | Pparg | 0.28 |
| 1247935 | origin recognition complex, subunit 4-like (S. cerevisiae) | Orc4l | 0.28 |
| 820373 | adaptor-related protein complex AP-3, mu 1 subunit | Ap3m1 | 0.28 |
| 851348 | molecule possessing ankyrin-repeats induced by lipopolysaccharide | Mail-pending | 0.28 |
| 891475 | ubiquitin-conjugating enzyme E2, J1 | Ube2j1 | 0.28 |
| 438183 | discoidin domain receptor family, member 2 | Ddr2 | 0.27 |
| 467172 | parathyroid hormone receptor | Pthr | 0.27 |
| 478472 | RAS p21 protein activator 3 | Rasa3 | 0.27 |
| 617529 | macrophage activation 2 | Mpa2 | 0.27 |
| 1265616 | angio-associated migratory protein | Aamp | 0.27 |
| 1054558 | electron transferring flavoprotein, alpha polypeptide | Etfa | 0.27 |
| 1246498 | transient receptor potential cation channel, subfamily M, member 1 | Trpm1 | 0.27 |
| 313840 | chondroitin 4-sulfotransferase 2 | C4st2-pending | 0.26 |
| 317958 | angiomotin like 2 | Amotl2 | 0.26 |
| 468210 | FMS-like tyrosine kinase 1 | Flt1 | 0.26 |
| 580715 | lymphocyte antigen 6 complex, locus A | Ly6 | 0.26 |
| 658233 | tumor necrosis factor receptor superfamily, member 19 | Tnfrsf19 | 0.26 |
| 831959 | phospholipase A2, activating protein | Plaa | 0.26 |
| 669953 | sialyltransferase 10 (alpha-2,3-sialyltransferase VI) | Siat10 | 0.26 |
| 697786 | resistin like beta | Retnlb | 0.26 |
| 735607 | cDNA sequence AF155546 | AF155546 | 0.26 |
| 777018 | selenoprotein P, plasma, 1 | Sepp1 | 0.26 |
| 851327 | macrophage galactose N-acetyl-galactosamine specific lectin 1 | Mgl1 | 0.26 |
| 875978 | mesenchyme homeobox 2 | Meox2 | 0.26 |
| 935316 | E26 avian leukemia oncogene 2, 3' domain | Ets2 | 0.26 |
| 1246561 | "a disintegrin-like and metalloprotease (reprolysin type) with thrombospondin type 1 motif, 2" |  | 0.26 |
| 334132 | procollagen, type VI, alpha 1 | Col6a1 | 0.25 |
| 404615 | benzodiazepine receptor, peripheral | Bzrp | 0.25 |
| 677488 | calcium binding protein P22 | Chp-pending | 0.25 |
| 1177632 | frizzled homolog 6 (Drosophila) | Fzd6 | 0.25 |
| 1532803 | mitogen activated protein kinase kinase kinase 1 | Map3k1 | 0.25 |
| 333498 | laminin, alpha 2 | Lama2 | 0.24 |
| 350336 | peroxisome biogenesis factor 16 | Pex16 | 0.24 |
| 419756 | erythrocyte protein band 7.2 | Epb7.2 | 0.24 |
| 466632 | chromobox homolog 5 (Drosophila HP1a) | Cbx5 | 0.24 |
| 581101 | phenylalanine hydroxylase | Pah | 0.24 |
| 597005 | fibronectin 1 | Fn1 | 0.24 |
| 676578 | degenerative spermatocyte homolog (Drosophila) | Degs | 0.24 |
| 692257 | matrix metalloproteinase 23 | Mmp23 | 0.24 |
| 809179 | multiple PDZ domain protein | Mpdz | 0.24 |
| 874658 | ATPase, H+ transporting, lysosomal V0 subunit a isoform 1 | Atp6v0a1 | 0.24 |
| 949509 | glutamyl aminopeptidase | Enpep | 0.24 |
| 1068748 | tetranectin (plasminogen binding protein) | Tna | 0.24 |
| 1247094 | synapse associated protein 1 | Syap1 | 0.24 |
| 1247529 | chloride channel 4-2 | Clcn4-2 | 0.24 |
| 1346958 | regulator of G-protein signaling 5 | Rgs5 | 0.24 |
| 313859 | diacylglycerol O-acyltransferase 1 | Dgat1 | 0.23 |
| 329372 | 2,4-dienoyl CoA reductase 1, mitochondrial | Decr1 | 0.23 |
| 338088 | enoyl coenzyme A hydratase 1, peroxisomal | Ech1 | 0.23 |
| 315082 | cathepsin L | Ctsl | 0.23 |
| 388288 | solute carrier family 25 (mitochondrial carrier; dicarboxylate transporter), member 10 | Slc25a10 | 0.23 |
| 385441 | 3-phosphoglycerate dehydrogenase |  | 0.23 |
| 478848 | discoidin domain receptor family, member 2 | Ddr2 | 0.23 |
| 776562 | hypertension related protein 1 | Mfn2 | 0.23 |
| 533003 | Stromal cell derived factor 1 | Sdf1 | 0.23 |
| 521951 | Williams-Beuren syndrome chromosome region 14 homolog (human) | Wbscr14 | 0.23 |
| 832055 | alpha thalassemia/mental retardation syndrome X-linked homolog (human) | Atrx | 0.23 |
| 876816 | leukotriene C4 synthase | Ltc4s | 0.23 |
| 874383 | lactotransferrin | Ltf | 0.23 |
| 1247911 | Ewing sarcoma homolog | Ewsh | 0.23 |
| 352450 | procollagen, type VI, alpha 1 | Col6a1 | 0.22 |
| 426965 | crystallin, alpha C | Cryac | 0.22 |
| 440103 | ectonucleotide pyrophosphatase/phosphodiesterase 2 | Enpp2 | 0.22 |
| 476163 | branched chain ketoacid dehydrogenase E1, beta polypeptide | Bckdhb | 0.22 |
| 583808 | guanylate nucleotide binding protein 2 | Gbp2 | 0.22 |
| 720736 | kelch-like 2, Mayven (Drosophila) | Klhl2 | 0.22 |
| 720566 | complement component 1, r subcomponent | C1r | 0.22 |
| 949551 | UDP-Gal:betaGlcNAc beta 1,3-galactosyltransferase, polypeptide 2 | B3galt2 | 0.22 |
| 1230508 | expressed sequence AW260363 | AW260363 | 0.22 |
| 403602 | histocompatibility 2, complement component factor B | H2-Bf | 0.21 |
| 483688 | 3-hydroxy-3-methylglutaryl-Coenzyme A synthase 1 | Hmgcs1 | 0.21 |
| 575700 | membrane bound C2 domain containing protein | Mbc2 | 0.21 |
| 620940 | nitric oxide synthase 3, endothelial cell | Nos3 | 0.21 |
| 1245994 | 2,4-dienoyl CoA reductase 1, mitochondrial | Decr1 | 0.21 |
| 1314769 | synuclein, gamma | Sncg | 0.21 |
| 330218 | dermatopontin | Dpt | 0.2 |
| 445565 | endothelial-specific receptor tyrosine kinase | Tek | 0.2 |
| 575665 | dual specificity phosphatase 1 | Dusp1 | 0.2 |
| 693315 | CD1d1 antigen | Cd1d1 | 0.2 |
| 692256 | cyclic AMP phosphoprotein, 19 kDa | Arpp19-pending | 0.2 |
| 777640 | complement component factor h | Cfh | 0.2 |
| 850067 | neuropilin | Nrp | 0.2 |
| 876418 | "ATP-binding cassette, sub-family D (ALD), member 4" |  | 0.2 |
| 1177891 | solute carrier family 24 (sodium/potassium/calcium exchanger), member 3 | Slc24a3 | 0.2 |
| 1230982 | RAS, dexamethasone-induced 1 | Rasd1 | 0.2 |
| 1244221 | nitric oxide synthase 3, endothelial cell | Nos3 | 0.2 |
| 1396458 | folate receptor 2 (fetal) | Folr2 | 0.2 |
| 1054503 | "nuclear receptor subfamily 1, group I, member 3" |  | 0.2 |
| 1177749 | pre T-cell antigen receptor alpha |  | 0.2 |
| 338152 | slit homolog 3 (Drosophila) | Slit3 | 0.19 |
| 406295 | serine (or cysteine) proteinase inhibitor, clade A, member 3G | Serpina3g | 0.19 |
| 421749 | prion protein | Prnp | 0.19 |
| 425855 | lymphocyte antigen 6 complex, locus C | Ly6c | .19 |
| 439383 | slit homolog 3 (Drosophila) | Slit3 | .19 |
| 483777 | diacylglycerol O-acyltransferase 1 | Dgat1 | .19 |
| 483775 | "ATPase, Na+/K+ transporting, alpha 2 polypeptide" |  | .19 |
| 597748 | "DNA segment, Chr 10, ERATO Doi 398, expressed" |  | .19 |
| 874758 | chemokine (C-X-C motif) ligand 9 | sycb9 | .19 |
| 717904 | endothelial cell-selective adhesion molecule | Esam-pending | .19 |
| 737055 | ADP-ribosyltransferase (NAD+; poly (ADP-ribose polymerase)-like 3 | Adprtl3 | .19 |
| 734688 | melanoma cell adhesion molecule | Mcam | .19 |
| 873982 | poly(A)-specific ribonuclease (deadenylation nuclease) | Parn | .19 |
| 332442 | brain protein 44-like | Brp44l | .18 |
| 406218 | sialyltransferase 10 (alpha-2,3-sialyltransferase VI) | Siat10 | .18 |
| 424402 | Wiskott-Aldrich syndrome-like (human) | Wasl | .18 |
| 480467 | aquaporin 1 | Aqp1 | .18 |
| 577422 | small chemokine (C-C motif) ligand 11 | Ccl11 | .18 |
| 617816 | complement component 1, r subcomponent | C1r | .18 |
| 935524 | regulator of G-protein signaling 5 | Rgs5 | .18 |
| 679617 | nuclear receptor subfamily 1, group H, member 3 | Nr1h3 | .18 |
| 832131 | ethanol induced 6 | Etohi6 | .18 |
| 875108 | growth hormone receptor | Ghr | .18 |
| 1397793 | RIKEN cDNA 0610011H20 gene | 0610011H20Rik | .18 |
| 403525 | breast cancer anti-estrogen resistance 3 | Bcar3 | .17 |
| 465403 | breast cancer anti-estrogen resistance 3 | Bcar3 | .17 |
| 738252 | CCAAT/enhancer binding protein (C/EBP), alpha | Cebpa | .17 |
| 874740 | guanine nucleotide binding protein, alpha inhibiting 1 | Gnai1 | .17 |
| 1195665 | lipin 1 | Lpin1 | .17 |
| 1177633 | high mobility group 20A | Hmg20a | .17 |
| 466280 | claudin 15 | Cldn15 | .16 |
| 575333 | ADP-ribosyltransferase (NAD+; poly (ADP-ribose polymerase)-like 3 | Adprtl3 | .16 |
| 596348 | aldehyde dehydrogenase family 1, subfamily A7 | Aldh1a7 | .16 |
| 693346 | 6-phosphofructo-2-kinase/fructose-2,6-biphosphatase 1 | Pfkfb1 | .16 |
| 820303 | cyclin-dependent kinase inhibitor 2C (p18, inhibits CDK4) | Cdkn2c | .16 |
| 831668 | retinol binding protein 7, cellular | Rbp7 | .16 |
| 1247493 | lectin, galactose binding, soluble 12 | Lgals12 | .16 |
| 1265897 | nuclear receptor subfamily 1, group H, member 3 | Nr1h3 | .16 |
| 1400942 | prostaglandin E receptor 4 (subtype EP4) | Ptger4 | .16 |
| 316237 | dipeptidase 1 (renal) | Dpep1 | .15 |
| 418633 | macrophage galactose N-acetyl-galactosamine specific lectin 1 | Mgl1 | .15 |
| 574792 | microsomal glutathione S-transferase 3 | Mgst3 | .15 |
| 581125 | G protein gamma 3 linked gene | Gng3lg | .15 |
| 949423 | acetyl-Coenzyme A synthetase 2 (ADP forming) | Acas2 | .15 |
| 750503 | ELK3, member of ETS oncogene family | Elk3 | .15 |
| 808000 | neuropilin | Nrp | .15 |
| 890764 | CD1d1 antigen | Cd1d1 | .15 |
| 1179554 | caveolin 2 | Cav2 | .15 |
| 336159 | histocompatibility 2, complement component factor B | H2-Bf | .14 |
| 1066861 | 8-oxoguanine DNA-glycosylase 1 | Ogg1 | .14 |
| 1247547 | neuregulin 4 | Nrg4 | .14 |
| 318735 | ATPase, Ca++ transporting, cardiac muscle, fast twitch 1 | Atp2a1 | .13 |
| 427469 | ethanol induced gene product EIG180 | EIG180 | .13 |
| 619563 | solute carrier family 1, member 7 | Slc1a7 | .13 |
| 698175 | nidogen 1 | Nid1 | .13 |
| 747880 | acyl-Coenzyme A oxidase 1, palmitoyl | Acox1 | .13 |
| 746644 | lumican | Lum | .13 |
| 1382053 | myoneurin | Mynn | .13 |
| 332285 | carbonic anhydrase 4 | Car4 | .12 |
| 386417 | tensin | Tns | .12 |
| 582169 | procollagen C-endopeptidase enhancer 2 | Pcolce2 | .12 |
| 619934 | hypothetical protein LOC207728 | LOC207728 | .12 |
| 672972 | glutathione transferase zeta 1 (maleylacetoacetate isomerase) | Gstz1 | .12 |
| 948835 | cyclin-dependent kinase 7 (homolog of Xenopus MO15 cdk-activating kinase) | Cdk7 | .12 |
| 1195467 | fatty acid synthase | Fasn | .12 |
| 1179487 | immunoglobulin heavy chain 6 (heavy chain of IgM) | Igh-6 | .12 |
| 1245874 | solute carrier family 2 (facilitated glucose transporter), member 5 | Slc2a5 | .12 |
| 1365243 | plectin 1 | Plec1 | .12 |
| 455945 | hydroxysteroid 11-beta dehydrogenase 1 | Hsd11b1 | .11 |
| 581906 | lipin 1 | Lpin1 | .11 |
| 640046 | baculoviral IAP repeat-containing 2 | Birc2 | .11 |
| 638244 | tumor protein D52 | Tpd52 | .11 |
| 851569 | guanine nucleotide binding protein, alpha inhibiting 1 | Gnai1 | .11 |
| 850671 | lipase, hormone sensitive | Lipe | .11 |
| 1511643 | CD36 antigen | Cd36 | .11 |
| 313322 | insulin-like growth factor 1 | Igf1 | .1 |
| 351557 | cell death-inducing DNA fragmentation factor, alpha subunit-like effector A | Cidea | .1 |
| 419437 | nidogen 1 | Nid1 | .1 |
| 571367 | BCL2/adenovirus E1B 19 kDa-interacting protein 1, NIP3 | Bnip3 | .1 |
| 676164 | histocompatibility 2, complement component factor B | H2-Bf | .1 |
| 695105 | alcohol dehydrogenase 1 (class I) | Adh1 | .1 |
| 1248540 | solute carrier family 1, member 7 | Slc1a7 | .1 |
| 1247905 | chemokine (C-X-C motif) ligand 9 | scyb9 | .1 |
| 463388 | BCL2/adenovirus E1B 19 kDa-interacting protein 1, NIP3 | Bnip3 | .09 |
| 477066 | four and a half LIM domains 1 | Fhl1 | .09 |
| 681802 | glutamyl aminopeptidase | Enpep | .09 |
| 851134 | adiponutrin | Adpn | .09 |
| 1495974 | vascular Rab-GAP/TBC-containing | Vrp-pending | .09 |
| 353456 | early B-cell factor 1 | Ebf1 | .08 |
| 761622 | Tnfa-induced adipose-related protein | Tiarp-pending | .08 |
| 776426 | pleiotropic regulator 1, PRL1 homolog (Arabidopsis) | Plrg1 | .08 |
| 832584 | Fc receptor, IgG, low affinity III | Fcgr3 | .08 |
| 875530 | monoglyceride lipase | Mgll | .08 |
| 875422 | tissue inhibitor of metalloproteinase 4 | Timp4 | .08 |
| 1179017 | frizzled homolog 4 (Drosophila) | Fzd4 | .08 |
| 1195796 | nicotinamide N-methyltransferase | Nnmt | .08 |
| 1247470 | Tnfa-induced adipose-related protein | Tiarp-pending | .08 |
| 876373 | Fgd1 family, member 2 | Fgd2 | .08 |
| 403656 | sorbin and SH3 domain containing 1 | Sorbs1 | .07 |
| 439814 | early B-cell factor 1 | Ebf1 | .07 |
| 473778 | pyruvate carboxylase | Pcx | .07 |
| 576881 | fatty acid synthase | Fasn | .07 |
| 678863 | esterase 22 | Es22 | .07 |
| 695845 | integrin alpha 7 | Itga7 | .07 |
| 808829 | frizzled homolog 4 (Drosophila) | Fzd4 | .07 |
| 831701 | transcription factor 1 | Tcf1 | .07 |
| 850276 | insulin-like growth factor binding protein 6 | Igfbp6 | .07 |
| 851302 | doublesex and mab-3 related transcription factor 2 | Dmrt2 | .07 |
| 862840 | interferon concensus sequence binding protein | Icsbp | .07 |
| 1179233 | carbohydrate (keratan sulfate Gal-6) sulfotransferase 1 | Chst1 | .07 |
| 1246574 | angiopoietin-like 4 | Angptl4 | .07 |
| 638791 | mannosidase 1, alpha | Man1a | .06 |
| 820177 | amine oxidase, copper containing 3 | Aoc3 | .06 |
| 329741 | angiopoietin-like 4 | Angptl4 | .05 |
| 335220 | monoglyceride lipase | Mgll | .05 |
| 579349 | epoxide hydrolase 2, cytoplasmic | Ephx2 | .05 |
| 764542 | epoxide hydrolase 2, cytoplasmic | Ephx2 | .05 |
| 570675 | glycerol phosphate dehydrogenase 1, cytoplasmic adult | Gdc1 | .04 |
| 1314022 | lipoprotein lipase | Lpl | .04 |
| 331186 | caveolin, caveolae protein, 22 kDa | Cav | .03 |
| 596968 | caveolin, caveolae protein, 22 kDa | Cav | .03 |
| 864344 | monocyte to macrophage differentiation-associated | Mmd | .03 |
| 864409 | CD36 antigen | Cd36 | .03 |
| 672437 | mesenchyme homeobox 2 | Meox2 | .03 |
| 721051 | peroxisome proliferator activated receptor gamma | Pparg | .03 |
| 1248283 | cDNA sequence BC018222 | BC018222 | .03 |
| 948509 | caveolin, caveolae protein, 22 kDa | Cav | .03 |
| 949623 | UDP-Gal:betaGlcNAc beta 1,3-galactosyltransferase, polypeptide 2 | B3galt2 | .02 |
| 890915 | peroxisome proliferator activated receptor gamma | Pparg | .02 |
| 1068786 | DEAD/H (Asp-Glu-Ala-Asp/His) box polypeptide 50 | Ddx50 | .02 |
| 890429 | carboxylesterase 3 | Ces3 | .01 |
| 891203 | leptin | Lep | .01 |
| 831665 | fat specific gene 27 | Fsp27 | .01 |
| 832222 | fatty acid binding protein 4, adipocyte | Fabp4 | .01 |
| 850835 | small inducible cytokine subfamily B, member 15 | Scyb15 | .01 |
| 1247588 | adipocyte complement related protein of 30 kDa | Acrp30 | .01 |
| 1245404 | lipoprotein lipase | Lpl | .01 |
| 948648 | protein phosphatase 1, regulatory (inhibitor) subunit 7 | Ppp1r7 | 0.002 |
| 832109 | resistin | Retn | 0.005 |
| 862835 | short stature homeobox 2 | Shox2 | 0.004 |
| 851374 | stearoyl-Coenzyme A desaturase 1 | Scd1 | 0.004 |
| 1314739 | carbonic anhydrase 3 | Car3 | 0.003 |
| 1396418 | carbonic anhydrase 3 | Car3 | 0.003 |
| 1448821 | fatty acid binding protein 4, adipocyte | Fabp4 | 0.004 |

*The average expression value of fat tissues divided by that of mammary tumors from MMTV-Wnt-1 TG mice and MMTV-Neu TG mice. p=<0.001. ESTs and riken cDNAs were excluded.
