# Supplementary material for: Changes in gene expression during the development of mammary tumors in MMTV-Wnt-1 transgenic mice
Source: Genome Biol. 2005 Sep 30;6(10):R84. doi: 10.1186/gb-2005-6-10-r84 (PMC1257467; doi:10.1186/gb-2005-6-10-r84)
Supplement: Additional File 7 — A table listing genes differentially expressed between mammary tumors and hyperplastic mammary glands from MMTV-Wnt-1 transgenic mice [file gb-2005-6-10-r84-S7.doc]

| **Additional data file 7. List of genes differentially expressed between mammary tumors and hyperplastic mammary glands from MMTV-Wnt-1 TG mice** | | | |
| --- | --- | --- | --- |
| **Image ID** | **Gene Name** | **Symbol** | **Expression Ratio**** |
| 747101 | calpain 6 | Capn6 | 9.65 |
| 737041 | betaine-homocysteine methyltransferase 2 | Bhmt2 | 9 |
| 622822 | expressed sequence AU019331 | Veli3-pending | 8.8 |
| 313426 | catenin beta | Catnb | 8.75 |
| 423028 | procollagen, type XI, alpha 1 | Col11a1 | 8.37 |
| 747583 | NCK-associated protein 1 | Nckap1 | 8.27 |
| 719592 | GATA binding protein 2 | Gata2 | 6.06 |
| 572428 | cyclin D1 | Ccnd1 | 5.53 |
| 423596 | actinin alpha 4 | Actn4 | 4.82 |
| 680894 | glial cell line derived neurotrophic factor family receptor alpha 1 | Gfra1 | 4.74 |
| 331681 | 6-pyruvoyl-tetrahydropterin synthase/dimerization cofactor of hepatocyte nuclear factor 1 alpha (TCF1) | Pcbd | 4.69 |
| 1247541 | apolipoprotein D | Apod | 4.67 |
| 733882 | protein phosphatase 2 (formerly 2A), regulatory subunit A (PR 65), alpha isoform | Ppp2r1a | 4.6 |
| 722262 | wingless-related MMTV integration site 5A | Wnt5a | 4.44 |
| 633209 | Rab6, kinesin-like | Rab6kifl | 4.32 |
| 468019 | interleukin 17B | Il17b | 4.27 |
| 444918 | mini chromosome maintenance deficient 6 (S. cerevisiae) | Mcmd6 | 4.17 |
| 373716 | tumor-associated calcium signal transducer 2 | Tacstd2 | 4.16 |
| 482170 | four jointed box 1 (Drosophila) | Fjx1 | 4.13 |
| 734795 | coproporphyrinogen oxidase | Cpo | 4.12 |
| 482943 | odd-skipped related 1 (Drosophila) | Osr1 | 4.1 |
| 734810 | transient receptor potential cation channel, subfamily C, member 4 associated protein | Trrp4ap-pending | 4.08 |
| 677093 | C-terminal binding protein 1 | Ctbp1 | 4.01 |
| 735225 | eukaryotic translation elongation factor 1 alpha 1 | Eef1a1 | 3.98 |
| 778942 | activating transcription factor 2 | Atf2 | 3.95 |
| 737492 | secretory carrier membrane protein 3 | Scamp3 | 3.86 |
| 424686 | protein kinase C substrate 80K-H | Prkcsh | 3.84 |
| 440571 | signal transducer and activator of transcription 3 | Stat3 | 3.83 |
| 733780 | faciogenital dysplasia homolog | Fgd1 | 3.78 |
| 329780 | cadherin 3 | Cdh3 | 3.74 |
| 439239 | protein kinase C substrate 80K-H | Prkcsh | 3.7 |
| 523588 | nuclear receptor coactivator 1 | Ncoa1 | 3.68 |
| 535949 | ubiquitin-like 1 (sentrin) activating enzyme E1A | Uble1a | 3.67 |
| 1365439 | kit oncogene | Kit | 3.66 |
| 464497 | serine (or cysteine) proteinase inhibitor, clade E,  member 2 | Serpine2 | 3.63 |
| 355990 | chondroitin sulfate proteoglycan 2 | Cspg2 | 3.62 |
| 476431 | nerve growth factor receptor | Ngfr | 3.61 |
| 670890 | HLA-B associated transcript 2 | 3110039B05Rik | 3.6 |
| 418952 | insulin-like growth factor binding protein 5 | Igfbp5 | 3.59 |
| 523945 | developmentally regulated GTP binding protein 2 | Drg2 | 3.56 |
| 904738 | receptor (calcitonin) activity modifying protein 2 | Ramp2 | 3.51 |
| 695687 | calponin 2 | Cnn2 | 3.49 |
| 474107 | tumor necrosis factor receptor superfamily, member 19 | Tnfrsf19 | 3.48 |
| 573320 | eukaryotic translation initiation factor 2, subunit 3, structural gene Y-linked | Eif2s3y | 3.44 |
| 403035 | cellular retinoic acid binding protein II | Crabp2 | 3.39 |
| 621246 | interferon concensus sequence binding protein | Icsbp | 3.38 |
| 524304 | NS1-associated protein 1-like | Nsap1l-pending | 3.37 |
| 441695 | claudin 3 | Cldn3 | 3.3 |
| 573520 | small protein effector 1 of Cdc42 | Spec1-pending | 3.29 |
| 335572 | dihydropyrimidinase-like 3 | Dpysl3 | 3.27 |
| 820355 | eukaryotic translation initiation factor 3, subunit 7 (zeta, 66/67 kDa) | Eif3s7 | 3.25 |
| 476407 | protein phosphatase 4, catalytic subunit | Ppp4c | 3.24 |
| 419193 | karyopherin (importin) beta 1 | Impnb | 3.22 |
| 423349 | polypyrimidine tract binding protein 1 | Ptb | 3.2 |
| 330910 | fibrosin | Trim8 | 3.16 |
| 333325 | tubulin, beta 5 | Tubb5 | 3.16 |
| 576166 | cyclin B1 | Ccnb1-rs1 | 3.08 |
| 638454 | solute carrier family 25 (mitochondrial deoxynucleotide carrier), member 19 | 2900089E13Rik | 3.08 |
| 1067302 | serologically defined colon cancer antigen 33 like | Sdccag33l-pending | 3.08 |
| 747427 | casein kinase II, alpha 1 related sequence 4 | Csnk2a1-rs4 | 3.06 |
| 468792 | cell division cycle 2 homolog A (S. pombe) | Cdc2a | 3.04 |
| 734780 | protein kinase, AMP-activated, gamma 1 non-catalytic subunit | Prkag1 | 3.04 |
| 536306 | procollagen, type I, alpha 1 | Col1a1 | 3.04 |
| 406897 | retinol binding protein 1, cellular | Rbp1 | 3.02 |
| 694987 | serine (or cysteine) proteinase inhibitor, clade E, member 2 | Serpine2 | 3.02 |
| 1244672 | ets homologous factor | Ehf | 3.02 |
| 876655 | EGL nine homolog 3 (C. elegans) | Egln3 | 3.01 |
| 736299 | staufen (RNA binding protein) homolog 1 (Drosophila) | Stau1 | 3.01 |
| 353456* | early B-cell factor 1 | Ebf | 0.33 |
| 596470 | CD79B antigen | Cd79b | 0.33 |
| 616709 | membrane-spanning 4-domains, subfamily A, member 1 | Ms4a2 | 0.33 |
| 672972* | glutathione transferase zeta 1 (maleylacetoacetate isomerase) | Gstz1 | 0.33 |
| 777640* | complement component factor h | Cfh | 0.33 |
| 1245994* | 2,4-dienoyl CoA reductase 1, mitochondrial | 1200012F07Rik | 0.33 |
| 1177749* | pre T-cell antigen receptor alpha | Ptcra | 0.33 |
| 480219 | Down syndrome critical region gene 1-like 1 | Dscr1l1 | 0.32 |
| 521951* | Williams-Beuren syndrome chromosome region 14 homolog (human) | Wbscr14 | 0.32 |
| 775253 | A kinase (PRKA) anchor protein (gravin) 12 | Akap12 | 0.31 |
| 351420 | sarcoglycan, gamma (35kD dystrophin-associated glycoprotein) | Sgcg | 0.31 |
| 403656* | sorbin and SH3 domain containing 1 | Sh3d5 | 0.31 |
| 596968* | caveolin, caveolae protein, 22 kDa | Cav | 0.31 |
| 679896* | dipeptidylpeptidase 4 | Dpp4 | 0.31 |
| 831701* | transcription factor 1 | Tcf1 | 0.31 |
| 1195467* | fatty acid synthase | Fasn | 0.31 |
| 387449 | glucocorticoid-induced leucine zipper | Gilz | 0.3 |
| 466632* | chromobox homolog 5 (Drosophila HP1a) | Cbx5 | 0.3 |
| 479895* | platelet derived growth factor receptor, alpha polypeptide | Pdgfra | 0.3 |
| 949423* | acetyl-Coenzyme A synthetase 2 (ADP forming) | Acas1 | 0.3 |
| 876373* | Fgd1 family, member 2 | Fgd2 | 0.3 |
| 334182 | amyotrophic lateral sclerosis 2 (juvenile) homolog (human) | 3222402C23Rik | 0.29 |
| 331186* | caveolin, caveolae protein, 22 kDa | Cav | 0.29 |
| 466191 | calsequestrin 2 | Casq2 | 0.29 |
| 571367* | BCL2/adenovirus E1B 19 kDa-interacting protein 1, NIP3 | Bnip3 | 0.29 |
| 493675 | actinin alpha 3 | Actn3 | 0.29 |
| 621878 | selectin, lymphocyte | Sell | 0.29 |
| 891203* | leptin | Lep | 0.29 |
| 678523 | hemolytic complement | Hc | 0.29 |
| 1381935* | histocompatibility 13 | H13 | 0.29 |
| 402053 | protein phosphatase 1, regulatory (inhibitor) subunit 1A | Ppp1r1a | 0.28 |
| 420322* | procollagen, type III, alpha 1 | Col3a1 | 0.28 |
| 406218* | sialyltransferase 10 (alpha-2,3-sialyltransferase VI) | Siat10 | 0.28 |
| 581906* | lipin 1 | Lpin1 | 0.28 |
| 676164* | histocompatibility 2, complement component factor B | H2-Bf | 0.28 |
| 1365243* | plectin 1 | Plec1 | 0.28 |
| 313322* | insulin-like growth factor 1 | Igf1 | 0.27 |
| 333498* | laminin, alpha 2 | Lama2 | 0.27 |
| 440344 | glutamine synthetase | Glns | 0.27 |
| 463388* | BCL2/adenovirus E1B 19 kDa-interacting protein 1, NIP3 | Bnip3 | 0.27 |
| 598468 | ryanodine receptor 1, skeletal muscle | Ryr1 | 0.27 |
| 332442* | brain protein 44-like | Brp44l | 0.26 |
| 621166 | ectonucleotide pyrophosphatase/phosphodiesterase 2 | Enpp2 | 0.26 |
| 622274 | membrane-spanning 4-domains, subfamily A, member 1 | Ms4a2 | 0.26 |
| 761622* | Tnfa-induced adipose-related protein | Tiarp-pending | 0.26 |
| 374030 | myosin binding protein H | Mybph | 0.25 |
| 864344* | monocyte to macrophage differentiation-associated | Mmd | 0.25 |
| 681802* | glutamyl aminopeptidase | Enpep | 0.25 |
| 747038 | procollagen, type III, alpha 1 | Col3a1 | 0.25 |
| 386901 | myelin protein zero | Mpz | 0.24 |
| 618572 | interferon activated gene 203 | Ifi203 | 0.24 |
| 617816* | complement component 1, r subcomponent | C1r | 0.24 |
| 638791* | mannosidase 1, alpha | Man1a | 0.24 |
| 862840* | interferon concensus sequence binding protein | Icsbp | 0.24 |
| 1179487* | immunoglobulin heavy chain 6 (heavy chain of IgM) | Igh-6 | 0.23 |
| 1195907* | Z-DNA binding protein 1 | Dlm1-pending | 0.23 |
| 330218* | dermatopontin | Dpt | 0.22 |
| 579349* | epoxide hydrolase 2, cytoplasmic | Ephx2 | 0.22 |
| 336726 | LIM domain binding 3 | Ldb3 | 0.21 |
| 473778* | pyruvate carboxylase | Pcx | 0.21 |
| 570675* | glycerol phosphate dehydrogenase 1, cytoplasmic adult | Gdc1 | 0.21 |
| 850276* | insulin-like growth factor binding protein 6 | Igfbp6 | 0.21 |
| 422161 | ryanodine receptor 1, skeletal muscle | Ryr1 | 0.2 |
| 721051* | peroxisome proliferator activated receptor gamma | Pparg | 0.2 |
| 315787 | upregulated during skeletal muscle growth 4 | Usmg4 | 0.19 |
| 426741 | myozenin 1 | Myoz | 0.19 |
| 949623* | UDP-Gal:betaGlcNAc beta 1,3-galactosyltransferase, polypeptide 2 | B3galt2 | 0.19 |
| 735605 | platelet derived growth factor receptor, alpha polypeptide | Pdgfra | 0.19 |
| 318735* | ATPase, Ca++ transporting, cardiac muscle, fast twitch 1 | Atp2a1 | 0.18 |
| 492441 | myelin protein zero | Mpz | 0.18 |
| 948509* | caveolin, caveolae protein, 22 kDa | Cav | 0.18 |
| 351557* | cell death-inducing DNA fragmentation factor, alpha subunit-like effector A | Cidea | 0.17 |
| 483775* | ATPase, Na+/K+ transporting, alpha 2 polypeptide | Atp1a2 | 0.17 |
| 890915* | peroxisome proliferator activated receptor gamma | Pparg | 0.17 |
| 697786* | resistin like beta | Retnlb | 0.17 |
| 832584* | Fc receptor, IgG, low affinity III | Fcgr3 | 0.16 |
| 1314022* | lipoprotein lipase | Lpl | 0.16 |
| 477066* | four and a half LIM domains 1 | Fhl1 | 0.15 |
| 582169* | procollagen C-endopeptidase enhancer 2 | 2400001O18Rik | 0.14 |
| 695105* | alcohol dehydrogenase 1 (class I) | Adh1 | 0.14 |
| 891510 | myozenin 1 | Myoz | 0.14 |
| 891094 | titin immunoglobulin domain protein (myotilin) | Ttid | 0.13 |
| 316237* | dipeptidase 1 (renal) | Dpep1 | 0.12 |
| 641512* | neutrophil cytosolic factor 4 | Ncf4 | 0.12 |
| 746644* | lumican | Lum | 0.12 |
| 455945* | hydroxysteroid 11-beta dehydrogenase 1 | Hsd11b1 | 0.11 |
| 764542* | epoxide hydrolase 2, cytoplasmic | Ephx2 | 0.11 |
| 1068786* | nucleolar protein GU2 | GU2 | 0.11 |
| 317868 | chloride intracellular channel 4 (mitochondrial) | Clic4 | 0.1 |
| 402105 | secretogranin III | Scg3 | 0.1 |
| 439638 | myosin, heavy polypeptide 3, skeletal muscle, embryonic | Myh3 | 0.1 |
| 335883 | paraoxonase 1 | Pon1 | 0.08 |
| 425653 | mast cell protease 5 | Mcpt5 | 0.08 |
| 831665* | fat specific gene 27 | Fsp27 | 0.07 |
| 1247588* | adipocyte complement related protein of 30 kDa | Acrp30 | 0.07 |
| 1348345* | follistatin-like 3 | Fstl3 | 0.07 |
| 820307* | expressed sequence AI158848 | AI158848 | 0.07 |
| 948437* | expressed sequence C78977 | Mrpl9 | 0.07 |
| 334236 | muscle glycogen phosphorylase | Pygm | 0.06 |
| 864409* | CD36 antigen | Cd36 | 0.06 |
| 850835* | small inducible cytokine subfamily B, member 15 | Scyb15 | 0.06 |
| 1247485 | myosin, heavy polypeptide 4, skeletal muscle | Myh4 | 0.06 |
| 1245404* | lipoprotein lipase | Lpl | 0.06 |
| 832109* | resistin | Retn | 0.05 |
| 1448821* | fatty acid binding protein 4, adipocyte | Fabp4 | 0.05 |
| 466382 | myosin light chain, phosphorylatable, fast skeletal muscle | Mylpf | 0.04 |
| 479354 | troponin C, fast skeletal | Tncs | 0.04 |
| 890429* | carboxylesterase 3 | Ces3 | 0.04 |
| 832222* | fatty acid binding protein 4, adipocyte | Fabp4 | 0.03 |
| 862835* | short stature homeobox 2 | Shox2 | 0.03 |
| 851374* | stearoyl-Coenzyme A desaturase 1 | Scd1 | 0.02 |
| 1314739* | carbonic anhydrase 3 | Car3 | 0.02 |
| 1396418* | carbonic anhydrase 3 | Car3 | 0.02 |
| 1383598* | expressed sequence AI642080 | Hnrph1 | 0.02 |
| 948648* | protein phosphatase 1, regulatory (inhibitor) subunit 7 | Ppp1r7 | 0.01 |
| 776007 | major urinary protein 2 | Mup2 | 0.01 |

*Genes expressed at least 3-fold more (p=< 0.001) in fat tissues than in mammary tumors from both MMTV-Wnt-1 and MMTV-Neu TG mice.

**The average expression value of tumors from MMTV-Wnt-1 TG mice divided by that of hyperplastic mammary glands from MMTV-Wnt-1 TG mice. p=<0.001. ESTs and riken cDNAs were excluded.
